# Evaluating MIR and NIR Spectroscopy Coupled with Multivariate Analysis for Detection and Quantification of Additives in Tobacco Products

**DOI:** 10.3390/s24217018

**Published:** 2024-10-31

**Authors:** Zeb Akhtar, Michaël Canfyn, Céline Vanhee, Cédric Delporte, Erwin Adams, Eric Deconinck

**Affiliations:** 1 Scientific Direction Chemical and Physical Health Risks, Service of Medicines and Health Products, Sciensano, Rue Juliette Wytsmanstraat 14, B-1050 Brussels, Belgium; zeb.akhtar@sciensano.be (Z.A.); michael.canfyn@sciensano.be (M.C.); celine.vanhee@sciensano.be (C.V.); 2 Department of Pharmaceutical and Pharmacological Sciences, Pharmaceutical Analysis, KU Leuven, Herestraat 49, O&N2, PB 923, B-3000 Leuven, Belgium; erwin.adams@kuleuven.be; 3 RD3-Pharmacognosy, Bioanalysis and Drug Discovery Unit, Faculty of Pharmacy, Université Libre de Bruxelles (ULB), Bld Triomphe, Campus Plaine, CP 205/5, B-1050 Brussels, Belgium; cedric.delporte@ulb.ac.be; 4 Analytical Platform of the Faculty of Pharmacy, Faculty of Pharmacy, Université Libre de Bruxelles (ULB), Bld Triomphe, Campus Plaine, CP 205/5, B-1050 Brussels, Belgium

**Keywords:** tobacco products, MIR/NIR spectroscopy, multivariate calibration techniques, data analysis

## Abstract

The detection and quantification of additives in tobacco products are critical for ensuring consumer safety and compliance with regulatory standards. Traditional analytical techniques, like gas chromatography–mass spectrometry (GC–MS), liquid chromatography–mass spectrometry (LC–MS), and others, although effective, suffer from drawbacks, including complex sample preparation, high costs, lengthy analysis times, and the requirement for skilled operators. This study addresses these challenges by evaluating the efficacy of mid-infrared (MIR) spectroscopy and near-IR (NIR) spectroscopy, coupled with multivariate analysis, as potential solutions for the detection and quantification of additives in tobacco products. So, a representative set of tobacco products was selected and spiked with the targeted additives, namely caffeine, menthol, glycerol, and cocoa. Multivariate analysis of MIR and NIR spectra consisted of principal component analysis (PCA), hierarchical clustering analysis (HCA), partial least squares-discriminant analysis (PLS-DA) and soft independent modeling of class analogy (SIMCA) to classify samples based on targeted additives. Based on the unsupervised techniques (PCA and HCA), a distinction could be made between spiked and non-spiked samples for all four targeted additives based on both MIR and NIR spectral data. During supervised analysis, SIMCA achieved 87–100% classification accuracy for the different additives and for both spectroscopic techniques. PLS-DA models showed classification rates of 80% to 100%, also demonstrating robust performance. Regression studies, using PLS, showed that it is possible to effectively estimate the concentration levels of the targeted molecules. The results also highlight the necessity of optimizing data pretreatment for accurate quantification of the target additives. Overall, NIR spectroscopy combined with SIMCA provided the most accurate and robust classification models for all target molecules, indicating that it is the most effective single technique for this type of analysis. MIR, on the other hand, showed the overall best performance for quantitative estimation.

## 1. Introduction

Tobacco use in Europe began in the 16th century for mystical, social, and medical purposes, but its harmful health effects became evident by the mid-20th century, when smoking was linked to lung cancer. Despite the introduction of filtered cigarettes, addiction rates and lung cancer cases continued to rise, especially after industrial production and the widespread use of cigarettes during World War I [1,2,3,4]. Commercial cigarette brands contain not only fermented tobacco, paper, and filters, but also about 600 additives [5]. These additives serve various purposes. Humectants like glycerin and propylene glycol are added for moisture retention [6]. Sugars, cocoa, and licorice are added to replace what is lost of them during drying and to enhance flavor [7,8]. Casings, applied before drying, make up 1–5% of the tobacco’s weight, while individual flavor compounds (toppings) account for about 0.1% [9]. In addition to improving taste and manufacturing, additives can also increase the attractiveness, addictiveness, and toxicity of cigarettes.

The global tobacco epidemic led to the adoption of the WHO Framework Convention on Tobacco Control (FCTC) in 2003, which came into force in 2005 and was ratified by over 190 countries. This treaty aims to reduce global tobacco use through evidence-based and political measures. It includes strategies to curb both the demand and supply of tobacco. Article 9 requires countries to measure and regulate the content and emissions of tobacco products. Additionally, guidelines were established to reduce the attractiveness, addictiveness, and toxicity of tobacco, with attractiveness being the most detailed aspect so far. These guidelines were partially adopted by the European Commission in Directive 2014/40/EU [10,11].

The European Directive on the manufacturing, presentation, and sale of tobacco and related products was updated in April 2014 to reduce the attractiveness of tobacco products, particularly among children and adolescents, and to regulate nicotine-based electronic cigarettes for the first time. Key provisions include banning cigarettes and roll-your-own (RYO) tobacco with characterizing flavors, prohibiting additives that enhance toxicity, addictiveness, and attractiveness, and requiring manufacturers to inform authorities about the ingredients used. Additionally, the directive mandates that health warnings cover 65% of the front and back of packaging units. It further bans promotional labeling, ensures the traceability of tobacco products, requires notification to member states before introducing novel tobacco products, and regulates electronic cigarettes and refill containers [12].

Several studies have examined violations of legislation related to tobacco products [12,13,14,15], which is of interest for inspection services. However, there has been no quantitative analysis of such violations at the European level to date. This topic is crucial for consumer safety and is particularly relevant amid the ongoing debate over the safety of non-tobacco nicotine products, especially electronic cigarettes [16,17,18,19].

Recent studies have utilized various advanced techniques to detect additives and other molecules in tobacco smoking products. Gas chromatography–mass spectrometry (GC-MS) has been widely used for the comprehensive identification of volatile organic compounds in tobacco and tobacco smoke [20]. Liquid chromatography–mass spectrometry (LC-MS) was, for instance, used for the determination of coumarin and its additives [21], while mid-infrared spectroscopy (MIR) was used for the quantification of total nicotine in Algerian smokeless tobacco products [22]. For the latter, nuclear magnetic resonance (NMR) spectroscopy was also applied [23]. High-performance liquid chromatography (HPLC) with or without MS can be used for a whole range of products found in tobacco products [24], while inductively coupled plasma mass spectrometry (ICP-MS) and X-ray fluorescence (XRF) spectroscopy were applied to analyze heavy metals [25,26]. Thermogravimetric analysis (TGA) [27] and headspace solid-phase extraction [28] were specifically applied to study several additives, while pyrolysis-GC-MS was used to study aroma compounds and their behavior during combustion [29].

These techniques, although powerful for identifying specific compounds, have several drawbacks such as complex sample preparation, high cost, and lengthy analysis times. They may also miss broad classes of additives and require skilled operators. In contrast, MIR and NIR spectroscopy with multivariate analysis offer a rapid and non-destructive analysis of a wide range of compounds in tobacco products. They require less sample preparation, provide spatial mapping capabilities, and are generally more cost-effective.

In this paper, the efficacy of MIR and NIR spectroscopy coupled with multivariate analysis was explored for the rapid and comprehensive detection of four frequently encountered additives in tobacco products, i.e., caffeine, menthol, glycerol, and cocoa. After initial data exploration using unsupervised techniques, classification models were developed for the detection of the targeted compounds, followed by the calculation of regression models in order to estimate their concentration.

## 2. Materials and Methods

### 2.1. Tobacco Samples

A representative set of 12 tobacco products was selected and purchased from the market. Since products with the targeted additives were hard to find, the samples were split into different portions. Each commercial tobacco product was spiked with the different targeted additives in various concentrations. The product as such was considered as the negative or unspiked sample.

### 2.2. Reagents and Chemicals

Caffeine was purchased from Fagron (Nazareth, Belgium), methanol (99.9%) from Biosolve (Valkenswaard, The Netherlands), menthol (>99%) from Merck (Darmstadt, Germany), glycerol (99.5%) from Acros (Geel, Belgium), and cocoa from Nielson-Massey Vanillas (Waukegan, IL, USA).

### 2.3. Sample Preparation

The concentrations of various additives in tobacco were achieved by spiking tobacco samples with known quantities of the additives and measuring the levels. Each additive was considered separately, and all models made use of the spiked and the unspiked samples. There were in total 12 unspiked samples and 140 spiked samples. Menthol was spiked into tobacco at levels ranging from 1 to 5 mg/g [30]. To achieve this, a mortar and pestle were chilled in a refrigerator at 4 °C for 2.5 h. Following this, menthol and tobacco were precisely measured using an analytical weighing balance. The measured ingredients were then thoroughly mixed and homogenized using the chilled mortar and pestle for 1 min and immediately put in a vial (1 g/vial), which was sealed as soon as possible. Glycerol was spiked at concentrations from 2 to 5% of tobacco weight [6]. Cocoa was spiked into tobacco at concentrations from 1 to 20 mg/g [31]. For this process, cocoa and glycerol were dissolved in methanol to create a solution. The tobacco sample was then weighed, brought into contact with this solution, and vortexed to ensure thorough mixing. The sample was left at room temperature to allow the methanol to evaporate. Caffeine was spiked in small amounts after making a solution with methanol, typically around 0.1 to 1 mg/g [32]. Firstly, a precise amount of tobacco was carefully weighed. Next, known quantities of caffeine solution were added to this tobacco sample. Then, the tobacco sample was thoroughly vortexed and mixed to ensure uniform distribution of caffeine throughout. Following homogenization, the spiked tobacco samples were analyzed using chromatographic methods to check the correctness of the spiked concentrations. For caffeine, HPLC-UV was used; however, for menthol and glycerol, GC-FID was used. The parameters of the used methods are mentioned in Supplementary Table S1.

### 2.4. Data Acquisition

#### 2.4.1. MIR

**Figure 1:**
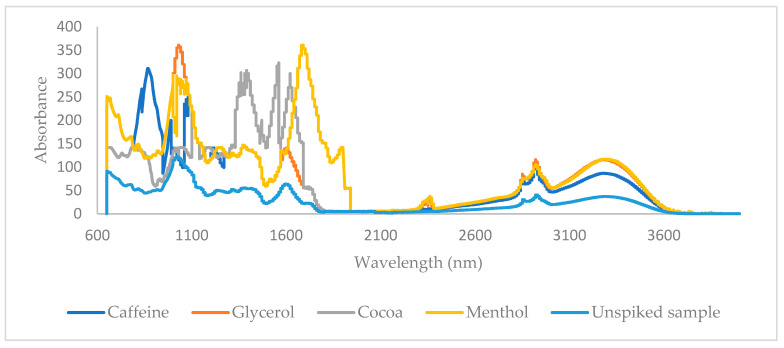
MIR data of a sample spiked with glycerol, menthol, caffeine, and cocoa and unspiked sample.

For the analysis, we utilized a Nicolet iS10 MIR spectrometer (ThermoFisher Scientific, Waltham, MA, USA) equipped with a Smart iTR accessory and a deuterated triglycine sulfate (DTGS) detector. The Smart iTR accessory features a single-bounce diamond crystal, which was calibrated weekly using a polystyrene film as a standard. We systematically recorded infrared (IR) spectra across the wavenumber range of 4000 to 400 cm^−1^ after setting up the equipment. Each spectrum was generated from 32 accumulated scans at a spectral resolution of 4 cm^−1^. Data processing was performed with OMNIC software version 8.3, developed by ThermoFisher Scientific (Madison, WI, USA). After data acquisition, the diamond crystal was thoroughly cleaned with a soft tissue soaked in methanol, followed by air-drying. Before analyzing each sample, we conducted blank measurements to evaluate the crystal’s potential contamination and carry-over effects, adhering to the strict protocols established by the European Directorate for the Quality of Medicines and HealthCare (EDQM) (2007) [33]. To ensure instrument accuracy, we recorded background spectra against ambient air on an hourly basis and included these data in our analysis. Ultimately, for each additive, we compiled a data matrix with dimensions of 38 × 6949, where 38 represents the total number of samples and 6949 corresponds to the number of wavenumbers used for chemometric analysis. The MIR spectra of a menthol-, caffeine-, glycerol-, and cocoa-spiked sample and the corresponding unspiked sample are shown in Figure 1.

#### 2.4.2. NIR

**Figure 2:**
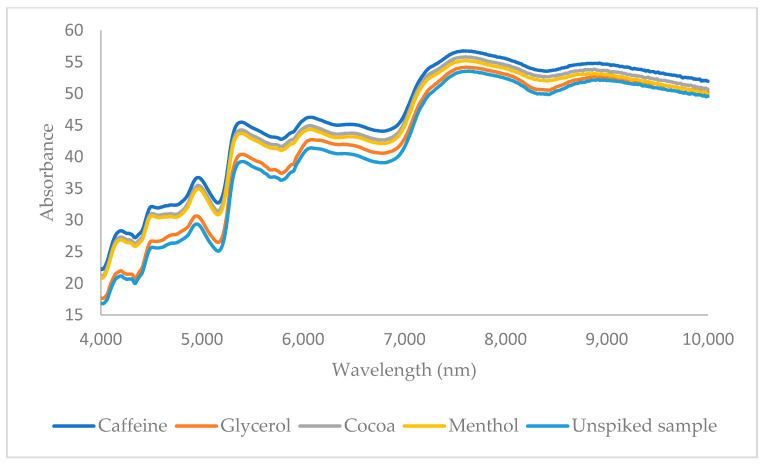
NIR data of a sample spiked with glycerol, menthol, caffeine, and cocoa and unspiked sample.

All samples were scanned using a Frontier MIR/NIR Spectrometer (PerkinElmer, Waltham, MA, USA) operating in reflectance mode with the NIR reflectance accessory. Spectra were acquired over the range of 10,000 to 4000 cm^−1^ with an 8 cm^−1^ resolution, averaging 16 scans per spectrum. Background spectra were collected using a diffuse reflector from Perkin Elmer between individual sample scans. Background subtraction and arithmetic corrections were applied to minimize background influences on the captured spectra. At the end, a data matrix with dimensions 38 × 1901 was obtained for each additive, where 38 is the total number of samples and 1901 the number of included wavenumbers for chemometric analysis. The NIR spectra of a spiked sample with menthol, caffeine, glycerol, and cocoa as well as the corresponding unspiked sample are shown in Figure 2.

#### 2.4.3. Data Preprocessing

In chemometrics for IR spectroscopy, data preprocessing involves several crucial steps, aimed at improving the quality and reliability of spectral data. Baseline correction was performed using the MIR and NIR (OMNIC software version 8.3) to mitigate baseline drift or curvature caused by instrumental or environmental factors, ensuring accurate representation of spectral features. For further analysis of the MIR data, only the fingerprint region was used (2000 to 650 cm^−1^). In NIR spectroscopy, wavelength range selection focused on pertinent regions of the spectrum containing significant chemical information specific to the analysis. Subsequent preprocessing steps were conducted using Matlab (MathWorks, Natick, MA, USA). Normalization techniques were applied to eliminate intensity variations resulting from sample concentration or instrumental effects, facilitating equitable comparisons between spectra. Autoscaling and signal noise variation (SNV) methods were explored in this context. Derivative transformations were employed to enhance spectral features, improve resolution of overlapping peaks, and enhance characterization and analysis [34,35]. Both SNV and derivative transformations are effective at reducing noise and artifacts, which is often crucial in spectroscopic data where measurements can be affected by external factors like light scattering, sample heterogeneity, or baseline drift. These techniques can enhance important signal characteristics (e.g., peaks or trends) and help isolate relevant information from confounding factors, improving model accuracy in subsequent data analysis. The selection of these methods is driven by their well-established efficacy in improving the interpretability and quality of spectroscopic measurements.

#### 2.4.4. Selection of Training and Test Sets

For model validation, selecting an external test set is crucial to assess the performance of the model. In this study, we employed the Duplex algorithm for this purpose. This algorithm ensures that the test set accurately represents the entire original dataset and is evenly distributed within the data space [35]. The Duplex algorithm operates by using Euclidean distances to identify sample pairs. It starts by finding the pair of samples with the maximum Euclidean distance between them, assigning it to the training set, and the next pair of samples with the highest Euclidean distance is assigned to the test set. This process continues by selecting additional pairs with the greatest distances until the desired number of samples is designated to the test set, while the remaining samples are added to the training set [35]. For robust model validation, approximately 20% of the total samples were designated to the external test set, ensuring that at least two unspiked samples were included. The remaining 80% were used to create the training set for model development.

#### 2.4.5. Principal Component Analysis

Principal component analysis (PCA) is a chemometric technique used to reduce the dimensionality of large datasets while preserving important information. It achieves this by transforming the original variables into a new set of orthogonal (uncorrelated) variables called principal components [36,37]. These components are ordered so that the first component explains the maximum variation in the dataset, the second component explains the maximum variance remaining after the first component is accounted for, and so on. PCA works by identifying patterns and correlations in the data and compressing the information into a smaller number of variables that retain as much of the original variance as possible. This reduction in dimensionality simplifies the dataset, making it easier to visualize and analyze while minimizing the loss of relevant information [37,38].

#### 2.4.6. Hierarchical Cluster Analysis

Hierarchical cluster analysis (HCA) is a clustering technique used to group similar objects into clusters based on their pairwise distances or similarities. HCA builds a tree-like hierarchical decomposition of the data, where clusters at each level of the hierarchy are formed by merging or splitting existing clusters. This method does not require a predefined number of clusters, allowing for flexibility in identifying structures within the data. HCA can be agglomerative, where each data point starts as its own cluster and is sequentially merged based on similarity, or divisive, where all data points begin in one cluster and are recursively split into smaller clusters [39]. HCA is widely used in various fields such as biology, the social sciences, and data mining for exploratory data analysis and pattern recognition tasks. For this study, HCA divisive clustering with Ward’s algorithm as similarity function was used.

#### 2.4.7. Soft Independent Modeling of Class Analogy

Soft independent modeling of class analogy (SIMCA) is a supervised classification technique that focuses on identifying similarities within classes rather than emphasizing the differences between them, a method known as disjoint class modeling. In SIMCA, each class is modeled separately using principal component analysis (PCA) [40]. The modeling process involves creating a defined space around the training samples of each class, which is characterized by two distance metrics: Euclidean distance to the SIMCA model and Mahalanobis distance within the score space [41]. The Euclidean distance assesses how closely a new sample’s projection corresponds to the SIMCA model of a given class, while the Mahalanobis distance accounts for correlations among variables and measures distances based on class covariance structures. When evaluating a new sample, its projection is compared to the established spaces surrounding each class’s training samples. If the projection resides within a class’s defined space, the sample is classified into that class. SIMCA is particularly effective for managing complex datasets with multiple classes, as it builds distinct models for each class, capturing the variability inherent to each and allowing for accurate predictions or classifications based on the proximity of new samples to existing class models [42,43].

#### 2.4.8. Partial Least Squares

Partial least squares (PLS) is a supervised projection method that shares similarities with principal component analysis (PCA). In PLS, latent variables are created as linear combinations of observed variables, with the aim of maximizing their covariance with a specific response variable. This technique is frequently employed in regression tasks where the response variable is continuous, such as dosage or concentration measurements. PLS-discriminant analysis (PLS-DA) is a variant specifically designed for classification tasks, enabling the analysis of categorical response variables and serving as an effective classification approach. PLS-DA is widely utilized in pattern recognition and various classification challenges [44,45,46].

### 2.5. Software for Data Analysis

All data processing in this study utilized Matlab version R2019b for scientific and numerical computing. The SIMCA and PLS algorithms were implemented using the ChemoAC toolbox version 4.1 developed by the ChemoAC Consortium (Brussels, Belgium).

## 3. Results and Discussion

MIR and NIR spectra were obtained for all tobacco samples. Before performing PCA, data preprocessing methods such as auto scaling, SNV, and first and second derivatives were applied to optimize the PCA results and improve data interpretation. The target molecules analyzed were menthol (a banned additive in the EU), caffeine (also a banned substance in EU, due to its stimulant properties), cocoa (used as a flavor enhancer), and glycerol (used as a humectant). They were selected for their public health and legal significance.

### 3.1. Unsupervised Classification Models

#### 3.1.1. PCA

**Figure 3:**
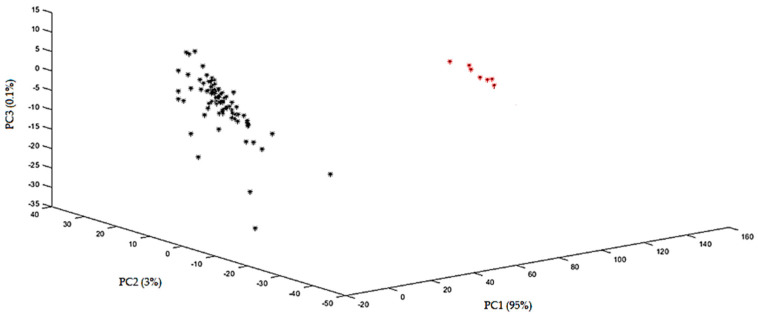
PCA plot obtained with the MIR spectra using the second derivative for menthol. Samples indicated with red stars are non-spiked samples, and samples indicated with black stars are spiked samples.

**Figure 4:**
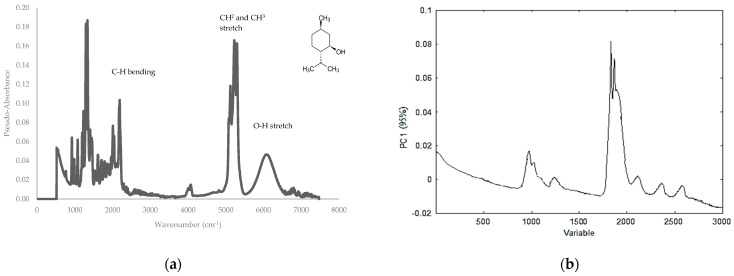
(**a**) MIR spectrum of menthol and (**b**) loadings on PC1 highlighting the region important for discrimination for menthol.

PCA score plots were obtained using the various data preprocessing techniques. The plots were evaluated for their ability to differentiate between spiked and non-spiked samples for each targeted additive separately. The optimal score plot for the distinction of menthol-containing samples in MIR was achieved using the second derivative (Figure 3). The first three principal components (PCs) accounted for over 98% of the total variance (PC1 = 95.0%, PC2 = 3.0%, and PC3 = 0.1%). The PCA score plots for caffeine, glycerol, and cocoa are shown in the Supplementary Data (Figures S1–S3). For caffeine, the score plot obtained after autoscaling, followed by the second derivative, showed the best separation, capturing over 99% of the total variance (PC1 = 98.0%, PC2 = 1.1%, and PC3 = 0.1%). For glycerol, the best score plot was obtained with the first derivative explaining 86% of the variance (PC1 = 77.0%, PC2 = 5.0%, and PC3 = 4.0%), and for cocoa, the best distinction could be made using SNV, including 97% of the total variance (PC1 = 92.0%, PC2 = 3.0%, and PC3= 2.0%). In general, PCA was able to differentiate between the spiked and non-spiked samples for all additives, based on MIR spectral data. According to the loadings on PC1, the region important for discrimination for menthol is 1800–2000 cm^−1^ (Figure 4), for caffeine 750–1000 cm^−1^, and for glycerol 1000–1150 cm^−1^ and for cocoa, the regions are 1650–1850 cm^−1^ and 1400–1600 cm^−1^ and they correspond to the characteristic regions in the spectrum of the pure additives. This proves that these regions could be identified as being responsible for the clustering. The MIR spectrum of menthol and loading plot on PC1 are shown in Figure 4a,b, respectively. Other spectra and loading plots on PC 1 for caffeine, glycerol, and cocoa are shown in the Supplementary Data (Figures S7–S9).

**Figure 5:**
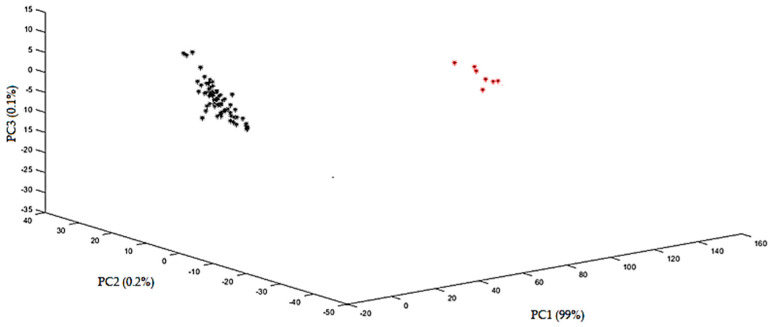
PCA plot obtained with the NIR spectra after autoscaling for menthol. Samples indicated with red stars are non-spiked samples, and samples indicated with black stars are spiked samples.

In NIR, the optimal score plot for menthol was achieved with autoscaling (Figure 5). The first three PCs explained over 99% of the total variance (PC1 = 99.0%, PC2 = 0.2%, and PC3 = 0.1%). The optimal score plots for caffeine, glycerol, and cocoa can be found in the Supplementary Material (Figures S4–S6). For caffeine, the optimal distinction was obtained with the second derivative, explaining over 98% of the total variance (PC1 = 97.0%, PC2 = 1.0%, and PC3 = 0.1%). For glycerol, the first derivative was calculated and PCA explained 95% of the total variance (PC1 = 88.0%, PC2 = 6.0%, and PC3 = 1.0%). For cocoa, the first derivative was taken too, and PCA captured nearly 98% of the total variance (PC1 = 90.0%, PC2 = 7.0%, and PC3 = 0.8%).

**Figure 6:**
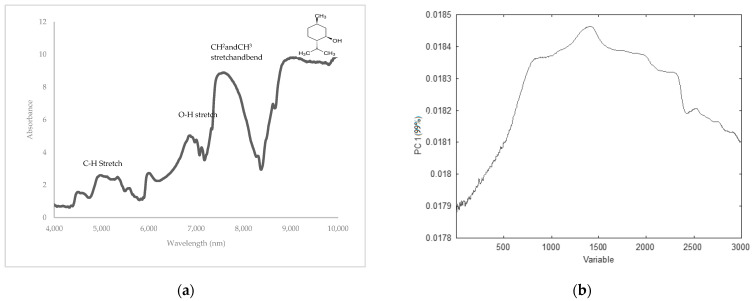
(**a**) NIR spectrum of menthol and (**b**) loadings on PC1 highlighting the region important for discrimination for menthol.

Overall, NIR was able to show good separations between the spiked samples and non-spiked samples for each of the targeted additives. After investigation of the loadings on PC1, no specific wavelengths or wavelength ranges could be identified as being responsible for the clustering. The NIR spectrum of menthol and loadings on PC 1 are shown in Figure 6a,b, respectively. The other spectra for caffeine, glycerol, and cocoa and the corresponding loading plots on PC1 are shown in the Supplementary Data (Figures S10–S12).

#### 3.1.2. HCA

**Figure 7:**
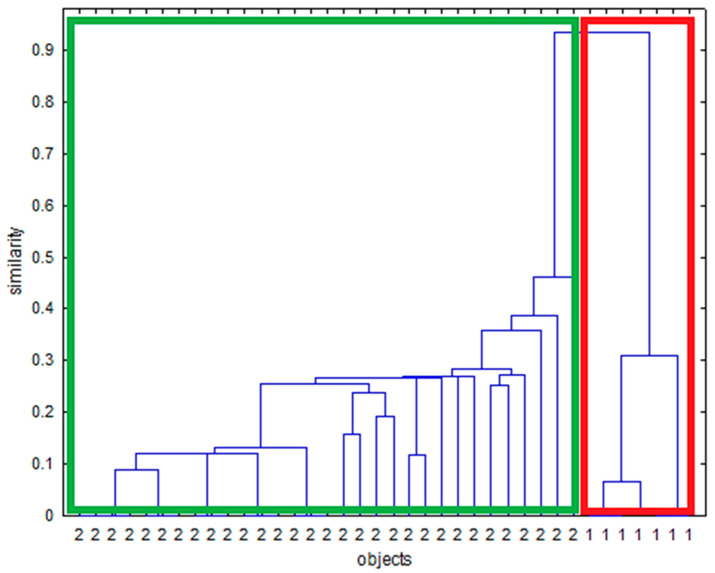
Dendrogram constructed via hierarchical clustering on MIR spectra for menthol. Samples indicated with 2 (green box) are spiked samples, and samples indicated with 1 (red box) are non-spiked samples.

**Figure 8:**
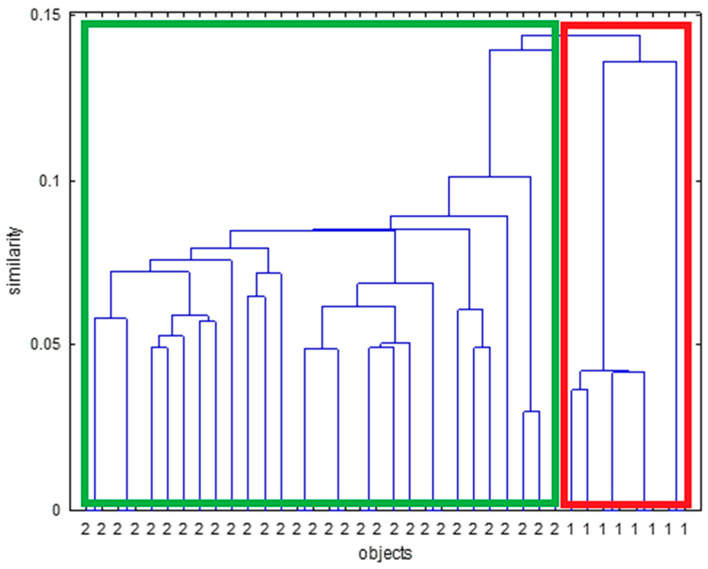
Dendrogram constructed via hierarchical clustering on NIR spectra for menthol. Samples indicated with 2 (green box) are spiked samples, and samples indicated with 1 (red box) are non-spiked samples.

For MIR spectroscopy, the HCA dendrogram for menthol (Figure 7) revealed two main clusters: one containing non-spiked samples and the other containing spiked samples. Similarly, for caffeine, glycerol, and cocoa, the HCA analysis effectively distinguished between spiked and non-spiked samples (Supplementary Figures S13–S18). Although for glycerol, it can be seen that the spiked samples are split into two clusters (Supplementary Figure S15). The second smaller cluster contains only spiked samples of the same original tobacco sample that seems to behave differently in MIR when spiked with glycerol. For NIR spectroscopy, the HCA dendrogram for menthol (Figure 8) showed two primary clusters: one for non-spiked samples and another for spiked samples. Similarly, caffeine, glycerol, and cocoa samples were also effectively differentiated into spiked and non-spiked groups using HCA (Supplementary Figures S13–S18). Also, here it can be seen that for glycerol, the spiked samples are split into two and that the spiked samples for one tobacco matrix behave slightly different in NIR (Supplementary Figure S16). The same phenomenon is observed for caffeine (Supplementary Figure S14).

### 3.2. Supervised Classification Models

Binary supervised classification models were developed using both SIMCA and PLS-DA to classify samples based on the presence of the additives. Four distinct models were developed, i.e., for menthol-containing samples, caffeine-containing samples, cocoa-containing samples, and glycerol-containing samples. The objective of these models was to accurately identify and differentiate samples according to the presence of these targeted molecules. Table 1, Table 2, Table 3 and Table 4 present the performance metrics of the best models obtained for each combination of spectroscopic technique, chemometric method, and target molecule, including their sensitivity, precision, and specificity.

#### 3.2.1. SIMCA

After preprocessing, SIMCA was used on both MIR and NIR data for each compound separately in binary modeling. The dataset was divided into a training and a test set. The training set was used to construct the models and for internal validation using 10-fold cross-validation. Afterward, the test set, kept separate from the training data, was used for external validation to ensure an unbiased evaluation of the models’ performance on new, unseen spectral data. This process confirmed the reliability and robustness of the models in predicting the presence of additives in unknown samples. The developed models allowed for identifying similarities and differences between the spectra of known classes, aiding in the classification of unknown samples based on their spectral characteristics. SIMCA, utilizing various data pretreatment methods, achieved classification accuracies between 88% and 100% for the external test sets and between 90% and 100% for cross-validation, as shown in Table 1. For caffeine detection using MIR spectroscopy with SNV data pretreatment, the best model resulted in one misclassified sample in both the external test set and cross-validation. In both cases, the misclassification was a false positive, as indicated by the specificity and precision values in Table 2. Sensitivity, specificity, and precision were used to evaluate the model’s performance. Sensitivity (true-positive rate) measures the proportion of actual positive cases correctly identified, ensuring that the test effectively detects true positives. Specificity (true-negative rate) assesses the test’s ability to correctly identify true negatives, minimizing false positives. Precision (positive predictive value) indicates the accuracy of positive results, showing how many of the predicted positive cases are truly positive. An analysis of the misclassified samples did not reveal a clear cause for the false positives, suggesting they may be due to random modeling errors. The focus remains on minimizing false negatives, as false positives can be verified by inspectors and confirmed in a laboratory. Using NIR spectroscopy with SNV pretreatment and the SIMCA technique for caffeine classification, both the external test set and cross-validation achieved no misclassifications.

For the glycerol samples, the most effective SIMCA model using autoscaled MIR spectra resulted in one false negative and two false positives during cross-validation, while the test set achieved no misclassifications. The high performance in external validation was reflected in the precision, specificity, and sensitivity metrics shown in Table 2. The misclassified sample during cross-validation had a glycerol concentration of 2% of the tobacco weight, while the two false positives indicated potential errors within the model. Misclassifications in the model can occur due to differences between the test samples and those used for training, known as deviations from the model. Alternatively, a misclassified sample might be an outlier due to spectral errors related to changes in settings or temperature variations. The misclassifications during cross-validation were likely due to deviations from the model. For glycerol classification using NIR spectroscopy with autoscaling pretreatment, one sample was misclassified in the test set, while during cross-validation, one false positive was found.

For menthol, MIR spectroscopy with first-derivative pretreatment led to one false positive in the external test set and one misclassified sample during cross-validation. The misclassification could not be conclusively explained and may be attributed to random modeling errors. In contrast, NIR spectroscopy with autoscaling and the SIMCA technique achieved no misclassifications in both the test set and cross-validation, demonstrating the model’s reliability.

For cocoa classification, using MIR spectroscopy with autoscaling pretreatment and SIMCA, the external test set had one false positive, while cross-validation showed two false negatives with concentrations ranging from 5 mg/g to 10 mg/g and one sample was misclassified as a false positive. These misclassifications are likely due to modeling errors. For NIR spectroscopy with autoscaling pretreatment, the model achieved perfect accuracy on the external test set, with one false positive during cross-validation, which could not be conclusively explained.

#### 3.2.2. PLS-DA Model

Subsequently, PLS-DA was utilized, which is a specialized form of PLS tailored for classification tasks, which enables the multivariate analysis technique to classify samples based on their spectral properties. The study assessed the classification performance of the various target molecules using both MIR and NIR spectroscopy, detailed in Table 3 and Table 4.

For caffeine, using MIR spectroscopy with first-derivative pretreatment, PLS-DA resulted in no misclassifications in both the external test set and cross-validation. Similarly, NIR spectroscopy with autoscaling pretreatment achieved perfect classification for both the external test set and cross-validation, with no misclassified samples.

For glycerol, MIR spectroscopy with autoscaling pretreatment also showed no misclassifications in the external test set, while cross-validation resulted in one sample misclassified as a false negative, with a concentration equivalent to 2% of tobacco weight and another sample was falsely identified as positive. Using NIR spectroscopy, the external test set had no misclassifications, but cross-validation showed two misclassified samples including one false negative associated with low concentrations (2% of tobacco weight) and one false positive, with the cause of the latter remaining uncertain.

For menthol assessment, MIR spectroscopy with second-derivative pretreatment achieved no misclassifications in the external test set, while cross-validation resulted in two false positives. For NIR spectroscopy with autoscaling pretreatment, the external test set had one false positive and cross-validation showed one false positive as well, with no clear explanation for the misclassification. For cocoa classification, using MIR spectroscopy with first-derivative pretreatment, the external test set had one false positive, while cross-validation resulted in two false positives and one false negative. NIR spectroscopy with first-derivative pretreatment achieved perfect classification in the external test set, but cross-validation showed six misclassifications: two false negatives and four false positives. The latter points at a possible robustness problem of the model. Among the misclassifications, two samples were incorrectly categorized as false negatives, with concentrations ranging from 2 mg/g to 10 mg/g and four samples were wrongly identified as false positives. Despite investigation, concrete explanations for these misclassifications remained elusive, suggesting potential modeling discrepancies.

Supplementary Figures S26–S33 show the loading plots, reflecting the importance of each variable on the first PLS factor. No specific regions could be identified as responsible for the modeling results, when compared to the specific regions in the respective spectra of the targeted additives.

### 3.3. Quantitative Models

**Figure 9:**
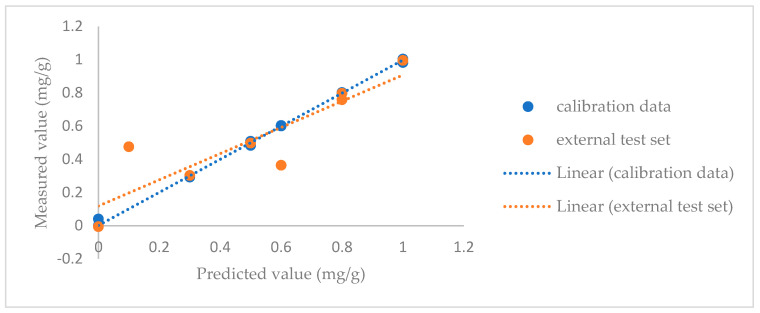
Insights into both the calibration accuracy and the predictive performance of the model for caffeine (model based on MIR spectra using the SNV as pretreatment method).

Caffeine and menthol are banned substances within the European Union, while glycerol and cocoa are for the moment still allowed in the majority of the member states, though their presence and concentration should be declared to the authorities. Given reported issues with sample conformity regarding the content of these additives and the fact that not all countries in the world have banned caffeine and menthol, the study explored the feasibility of constructing a quantitative PLS model based on MIR and NIR data to estimate their concentrations in tobacco. Quantification is also important to distinguish between trace levels or contaminations and intended addition, especially when it comes to seizures and legal procedures. Validation was again performed using both cross-validation and an external test set. The statistical parameters for quantification using the PLS model were evaluated using both MIR and NIR spectroscopy, coupled with various data pretreatment methods. Metrics including the root mean square error of calibration (RMSEC), the coefficient of determination for calibration (R^2^c), the root mean square error of prediction (RMSEP), the coefficient of determination for prediction (R²p), the root mean square error of cross-validation (RMSECV), and the coefficient of determination for cross-validation (R^2^cv) were assessed. These metrics provide insights into both the calibration accuracy and the predictive performance of the models as shown in Table 5. Figure 9 illustrates the caffeine calibration accuracy and the predictive performance. Similar figures for the other additives can be found in the Supplementary Data (Figures S19–S25). Figures S26–S33 in the Supplementary Data show the loading plots for the first PLS factor for the regression models. When comparing these to the spectra, no specific regions responsible for the modeling results could be identified.

For MIR spectroscopy, the caffeine model showed calibration values of R^2^c = 0.9983 and prediction values of R^2^p = 0.7781, with an RMSEP = 0.1514. The glycerol model exhibited an R^2^c = 0.7331 and an R^2^p = 0.6995, with an RMSEP = 0.1931. The menthol model displayed calibration values of R^2^c = 0.9582 and a prediction accuracy of R^2^p = 0.7710, with an RMSEP = 0.1601. For cocoa, the calibration accuracy was R^2^c = 0.8019, while the prediction accuracy was R^2^p = 0.7195, with an RMSEP = 0.2074.

In the case of NIR spectroscopy, the caffeine model demonstrated a calibration accuracy of R^2^c = 0.9983 and a prediction accuracy of R^2^p = 0.7138, with an RMSEP = 0.1453. The glycerol model showed an R^2^c = 0.8765 and an R^2^p = 0.6926, with an RMSEP = 0.2052. The menthol model achieved an R^2^c = 0.9443 and an R^2^p = 0.7572, with an RMSEP = 0.3119. For cocoa, the calibration accuracy was R^2^c = 0.7993, while the prediction accuracy was R^2^p = 0.6435, with an RMSEP = 0.2816.

Overall, it can be said that the quantitative models could be used to obtain a first estimation of the content of the targeted additives in the product, allowing to make a decision on the necessity for further analysis using other methods. It can give an estimation on the levels of concentration of glycerol and cocoa and make a definite distinction between trace amounts of caffeine and menthol and intended added doses.

## 4. Conclusions

MIR and NIR spectral data were collected for a set of spiked and non-spiked tobacco samples with four additives, namely, caffeine and menthol as banned substances and glycerol and cocoa that have to be declared. Following data preprocessing, an unsupervised analysis using both PCA and HCA showed that both techniques were able to give binary distinction between samples containing and not containing the respective targeted additives. Based on this initial data exploration, showing that the spectral differences can easily be linked to the presence of the additives, it was decided to proceed to supervised modeling for both classification and regression purposes.

For classification, SIMCA demonstrated robust classification performances, achieving accuracy rates ranging from 88% to 100% on the external test set and from 90% to 100% in cross-validation (Table 1). In general, it could be observed that the combination of NIR spectroscopy and SIMCA yielded better predictive models than the models based on MIR spectroscopy and SIMCA.

Also, PLS-DA modeling was explored, showing accuracy rates for the external test set between 88 and 100% and between 80 and 100% in cross-validation. In PLS-DA modeling, both spectroscopic techniques gave models with similar predictive performances.

Overall, NIR spectroscopy combined with SIMCA emerged as the preferred approach. Based on the results for the external test sets, one could emphasize that the four approaches, MIR-SIMCA, NIR-SIMCA, MIR-PLS-DA, and NIR-PLS-DA, give very comparable results, although NIR-SIMCA results in very good CCRs (correct classification rate) for both the test set and cross-validation. Especially the latter points are more robust and therefore more reliable models. This justifies the choice for NIR-SIMCA for the detection or classification of samples containing the targeted additives.

Regarding regression, both MIR and NIR spectroscopy, combined with suitable data pretreatment techniques such as SNV, autoscaling, and derivative transformations, can estimate the level of concentration of the target molecules. For regression, although close, MIR gave slightly better accuracy than NIR spectroscopy, mainly for the external test set. The quantitative estimations using spectroscopy should be seen as a first step and would allow inspection services to seize products and proceed to further analysis if deemed necessary. The presented approach can give a first idea about the conformity of the glycerol and cocoa contents compared to the declared contents and can differentiate between contamination with menthol and caffeine and intended addition.

It can be concluded that MIR and NIR spectroscopy can be used in the analysis of tobacco products for the presence of regulated additives. It was demonstrated that both additive detection and quantitative estimation are possible using basic chemometric techniques, like SIMCA and PLS(-DA), for caffeine, menthol, glycerol, and cocoa. This research also emphasizes that the selection of pretreatment and data analysis techniques is highly dependent on the matrix and the targeted molecule and should therefore be optimized for each additive separately.

## Figures and Tables

**Table 1:** Comprehensive overview of the performance of MIR and NIR, data pretreatment methods, and SIMCA in the classification of various target molecules.

Spectroscopic Technique	Data Pretreatment	Chemometric Technique	Target Molecule	No. of PCs	No. of Samples in External Test Set	Correct Classification Rate (External Test Set) [No. of Negative Samples]	No. of Samples in the Training Set	Correct Classification Rate (Cross-Validation) [No. of Negative Samples]
MIR	SNV		Caffeine	3-2	8	88% (7/8) [2/8]	30	97% (29/30) [5/30]
	Autoscaling		Glycerol	3-1	8	100% (8/8) [2/8]	30	90% (27/30) [5/30]
	1st derivative	SIMCA	Menthol	2-1	8	88% (7/8) [3/8]	30	97% (29/30) [4/30]
	Autoscaling		Cocoa	3-2	8	88% (7/8) [2/8]	30	90% (27/30) [5/30]
NIR	SNV		Caffeine	2-1	8	100% (8/8) [3/8]	30	100% (30/30) [4/30]
	Autoscaling		Glycerol	1-1	8	88% (7/8) [2/8]	30	97% (29/30) [5/30]
	Autoscaling	SIMCA	Menthol	2-1	8	100% (8/8) [2/8]	30	100% (30/30) [5/30]
	Autoscaling		Cocoa	2-2	8	100% (8/8) [3/8]	30	97% (29/30) [4/30]

**Table 2:** Classification statistics for cross-validation and test set for SIMCA model.

SIMCA												
MIR	Caffeine			Glycerol			Menthol			Cocoa		
	Precision	Specificity	Sensitivity	Precision	Specificity	Sensitivity	Precision	Specificity	Sensitivity	Precision	Specificity	Sensitivity
Cross-validation	0.96	0.85	1.00	0.92	0.75	0.66	0.96	0.85	1.00	0.96	0.85	0.66
Test set	0.87	0.50	1.00	1.00	1.00	1.00	0.87	0.50	1.00	0.87	0.50	1.00
NIR	Caffeine			Glycerol			Menthol			Cocoa		
	Precision	Specificity	Sensitivity	Precision	Specificity	Sensitivity	Precision	Specificity	Sensitivity	Precision	Specificity	Sensitivity
Cross-validation	1.00	1.00	1.00	0.96	0.85	1.00	1.00	1.00	1.00	0.96	0.85	1.00
Test set	1.00	1.00	1.00	0.87	0.50	1.00	1.00	1.00	1.00	1.00	1.00	1.00

**Table 3:** Comprehensive overview of the performance of MIR and NIR, data pretreatment methods, and PLS-DA in the classification of various target molecules.

Spectroscopic Technique	Data Pretreatment	Chemometric Technique	Target Molecule	No of Latent Variables	No of Samples in External Test Set	Correct Classification Rate (External Test Set) [No. of Negative Samples]	No of Samples in Training Set	Correct Classification Rate (Cross-Validation) [No. of Negative Samples]
MIR	1st derivative		Caffeine	4	8	100% (8/8) [3/8]	30	100% (30/30) [4/30]
	Autoscaling		Glycerol	2	8	100% (8/8) [2/8]	30	93% (28/30) [5/30]
	2nd derivative	PLS-DA	Menthol	7	8	100% (8/8) [3/8]	30	93% (28/30) [4/30]
	1st derivative		Cocoa	4	8	88% (7/8) [2/8]	30	90% (27/30) [5/30]
NIR	Autoscaling		Caffeine	2	8	100% (8/8) [2/8]	30	100% (30/30) [5/30]
	Autoscaling		Glycerol	6	8	100% (8/8) [2/8]	30	93% (28/30) [5/30]
	Autoscaling	PLS-DA	Menthol	4	8	88% (7/8) [2/8]	30	97% (29/30) [5/30]
	1st derivative		Cocoa	8	8	100% (8/8) [3/8]	30	80% (24/30) [4/30]

**Table 4:** Classification statistics for cross-validation and test set for PLS-DA model.

PLS-DA												
MIR	Caffeine			Glycerol			Menthol			Cocoa		
	Precision	Specificity	Sensitivity	Precision	Specificity	Sensitivity	Precision	Specificity	Sensitivity	Precision	Specificity	Sensitivity
Cross-validation	1.00	1.00	1.00	0.96	0.85	0.50	0.92	0.75	1.00	0.96	0.85	0.66
Test set	1.00	1.00	1.00	1.00	1.00	1.00	1.00	1.00	1.00	0.96	0.85	1.00
NIR	Caffeine			Glycerol			Menthol			Cocoa		
	Precision	Specificity	Sensitivity	Precision	Specificity	Sensitivity	Precision	Specificity	Sensitivity	Precision	Specificity	Sensitivity
Cross-validation	1.00	1.00	1.00	0.96	0.85	0.50	0.96	0.85	1.00	0.85	0.60	0.66
Test set	1.00	1.00	1.00	1.00	1.00	1.00	0.96	0.85	1.00	1.00	1.00	1.00

**Table 5:** Statistical parameters for quantification of the target molecules using the PLS model.

Spectroscopic Technique	Data Pretreatment	Target Molecule	No. of Latent Variables	RMSEC	R^2^c	RMSEP	R^2^p	RMSECV	R²cv
MIR	SNV	Caffeine	9	0.0088	0.9983	0.1514	0.7781	0.1634	0.7802
	2nd derivative	Glycerol	12	0.1541	0.7331	0.1931	0.6995	0.1923	0.7025
	1st derivative	Menthol	8	0.0670	0.9582	0.1601	0.7710	0.1580	0.7993
	1st derivative	Cocoa	8	0.1873	0.8019	0.2074	0.7195	0.1750	0.7584
NIR	1st derivative	Caffeine	10	0.0139	0.9983	0.1453	0.7138	0.1079	0.9141
	1st derivative	Glycerol	9	0.1078	0.8765	0.2052	0.6926	0.1700	0.7667
	Autoscaling	Menthol	13	0.0806	0.9443	0.3119	0.7572	0.1675	0.7738
	SNV	Cocoa	12	0.3112	0.7993	0.2816	0.6435	0.1900	0.7190

## Data Availability

The information presented in this research is not accessible to the general public because of certain limitations, such as concerns related to privacy and ethics.

## Supplementary Materials

The following supporting information can be downloaded at https://www.mdpi.com/article/10.3390/s24217018/s1, Figure S1: PCA plot obtained with the MIR spectra using autoscaling for Caffeine. Samples indicated with red stars are spiked samples and with black stars are non-spiked samples; Figure S2: PCA plot obtained with the MIR spectra using the first derivative for Glycerol. Samples indicated with red stars are spiked samples and with black stars are non-spiked samples; Figure S3: PCA plot obtained with the MIR spectra using SNV for Cocoa. Samples indicated with red stars are spiked samples and with black stars are non-spiked samples; Figure S4: PCA plot obtained with the NIR spectra using the Second derivative for Caffeine. Samples indicated with red stars are spiked samples and with black stars are non-spiked samples; Figure S5: PCA plot obtained with the NIR spectra using the first derivative for Glycerol. Samples indicated with red stars are spiked samples and with black stars are non-spiked samples; Figure S6: PCA plot obtained with the NIR spectra using the first derivative for Cocoa. Samples indicated with red stars are spiked samples and with black stars are non-spiked samples; Figure S7: (a) MIR spectrum of cocoa; (b) Loadings on PC1 highlighting the region important for discrimination for the cocoa; Figure S8: (a) MIR spectrum of glycerol; (b) Loadings on PC1 highlighting the region important for discrimination for the glycerol; Figure S9: (a) MIR spectrum of caffeine; (b) Loadings on PC1 highlighting the region important for discrimination for the caffeine; Figure S10: (a) NIR spectrum of cocoa; (b) Loadings on PC1 highlighting the region important for discrimination for the cocoa; Figure S11: (a) NIR spectrum of glycerol; (b) Loadings on PC1 highlighting the region important for discrimination for the glycerol; Figure S12: (a) NIR spectrum of caffeine; (b) Loadings on PC1 highlighting the region important for discrimination for the caffeine; Figure S13: Dendrogram constructed via hierarchical clustering on MIR spectra for Caffeine. Samples indicated with 2 (red box) are spiked samples and with 1 (green box) are non-spiked samples; Figure S14: Dendrogram constructed via hierarchical clustering on NIR spectra for Caffeine. Samples indicated with 2 (red box) are spiked samples, and with 1 (green box) are non-spiked samples; Figure S15: Dendrogram constructed via hierarchical clustering on MIR spectra for Glycerol. Samples indicated with 2 (red box) are spiked samples, and with 1 (green box) are non-spiked samples; Figure S16: Dendrogram constructed via hierarchical clustering on NIR spectra for Glycerol. Samples indicated with 2 (red box) are spiked samples, and with 1 (green box) are non-spiked samples; Figure S17: Dendrogram constructed via hierarchical clustering on MIR spectra for Cocoa. Samples indicated with 2 (red box) are spiked samples and with 1 (green box) are non-spiked samples; Figure S18: Dendrogram constructed via hierarchical clustering on NIR spectra for Cocoa. Samples indicated with 2 (red box) are spiked samples and with 1 (green box) are non-spiked samples; Figure S19: Insights into both the calibration accuracy and the predictive performance of the model for Cocoa (model based on MIR spectra using the 1st derivative as pretreatment method); Figure S20: Insights into both the calibration accuracy and the predictive performance of the model for Menthol (model based on MIR spectra using the 1st derivative as pretreatment method); Figure S21: Insights into both the calibration accuracy and the predictive performance of the model for Glycerol (model based on MIR spectra using the 2nd derivative as pretreatment method); Figure S22: Insights into both the calibration accuracy and the predictive performance of the model for Caffeine (model based on NIR spectra using the 1st derivative as pretreatment method); Figure S23: Insights into both the calibration accuracy and the predictive performance of the model for Cocoa (model based on NIR spectra using the SNV as pretreatment method); Figure S24: Insights into both the calibration accuracy and the predictive performance of the model for Glycerol (model based on NIR spectra using the 1st derivative as pretreatment method); Figure S25: Insights into both the calibration accuracy and the predictive performance of the model for Menthol (model based on NIR spectra using the autoscaling as pretreatment method); Figure S26: (a) PLS loading plot PLS1 for menthol (MIR) (b) PLS(DA) Loading plot PLS1 for menthol (MIR); Figure S27: (a) PLS loading plot PLS1 for caffeine (MIR) (b) PLS(DA) Loading plot PLS1 for caffeine (MIR); Figure S28: (a) PLS loading plot PLS1 for cocoa (MIR) (b) PLS(DA) Loading plot PLS1 for cocoa (MIR); Figure S29: (a) PLS loading plot PLS1 for glycerol (MIR) (b) PLS(DA) Loading plot PLS1 for glycerol (MIR); Figure S30: (a) PLS loading plot PLS1 for glycerol (NIR) (b) PLS(DA) Loading plot PLS1 for glycerol (NIR); Figure S31: (a) PLS loading plot PLS1 for cocoa (NIR) (b) PLS(DA) Loading plot PLS1 for cocoa (NIR); Figure S32: (a) PLS loading plot PLS1 for caffeine (NIR) (b) PLS(DA) Loading plot PLS1 for caffeine (NIR); Figure S33: (a) PLS loading plot PLS1 for menthol (NIR) (b) PLS(DA) Loading plot PLS1 for menthol (NIR); Table S1: GC-FID parameters to check the correctness of the spiked concentrations of menthol and glycerol for tobacco samples; Table S2: HPLC-UV parameters to check the correctness of the spiked concentrations of caffeine for tobacco samples.
