# Supplementary material for: Evaluating MIR and NIR Spectroscopy Coupled with Multivariate Analysis for Detection and Quantification of Additives in Tobacco Products
Source: Sensors (Basel). 2024 Oct 31;24(21):7018. doi: 10.3390/s24217018 (PMC11548177; doi:10.3390/s24217018)
Supplement: Supplementary file 1 [file sensors-24-07018-s001.zip › sensors-3224736-supplementary.pdf]

Supplementary data:

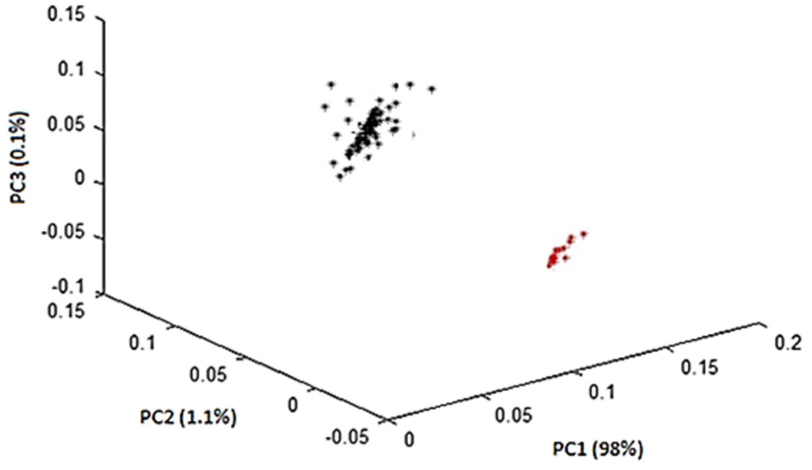

Figure S1. PCA plot obtained with the MIR spectra using autoscaling for Caffeine. Samples indicated with red stars are spiked samples and with black stars are non-spiked samples

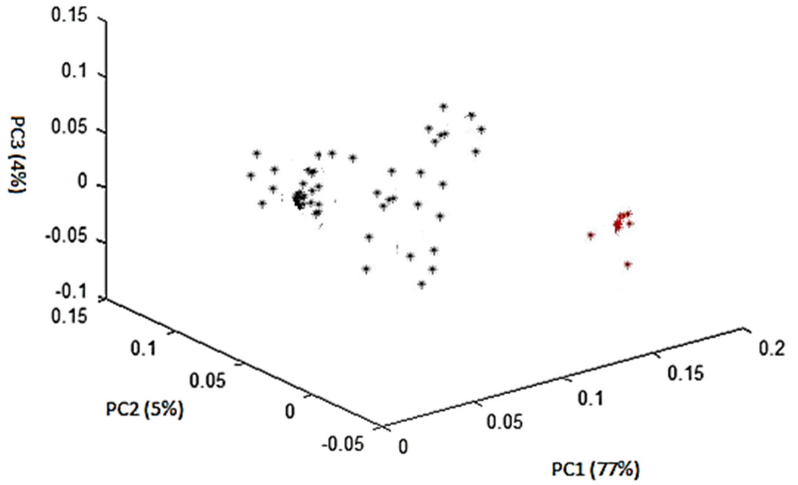

Figure S2. PCA plot obtained with the MIR spectra using the first derivative for Glycerol. Samples indicated with red stars are spiked samples and with black stars are non-spiked samples

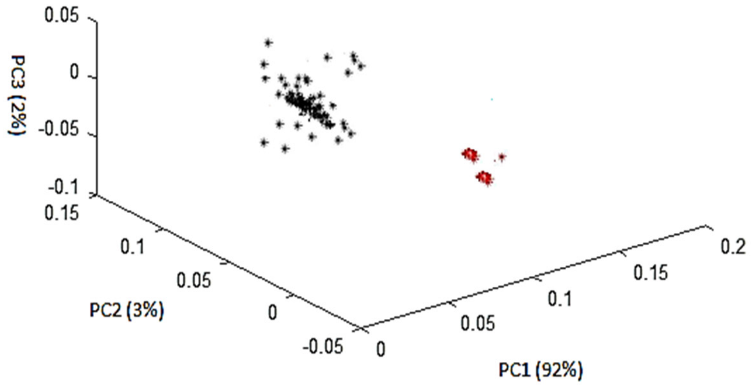

Figure S3. PCA plot obtained with the MIR spectra using SNV for Cocoa. Samples indicated with red stars are spiked samples and with black stars are non-spiked samples

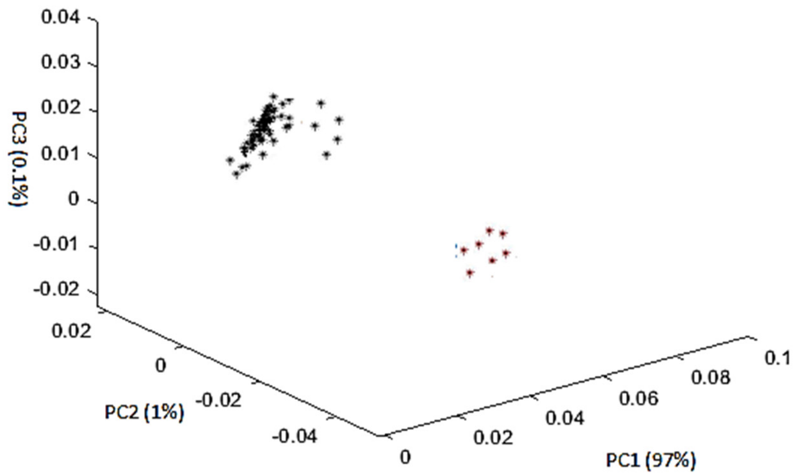

Figure S4. PCA plot obtained with the NIR spectra using the Second derivative for Caffeine. Samples indicated with red stars are spiked samples and with black stars are non-spiked samples

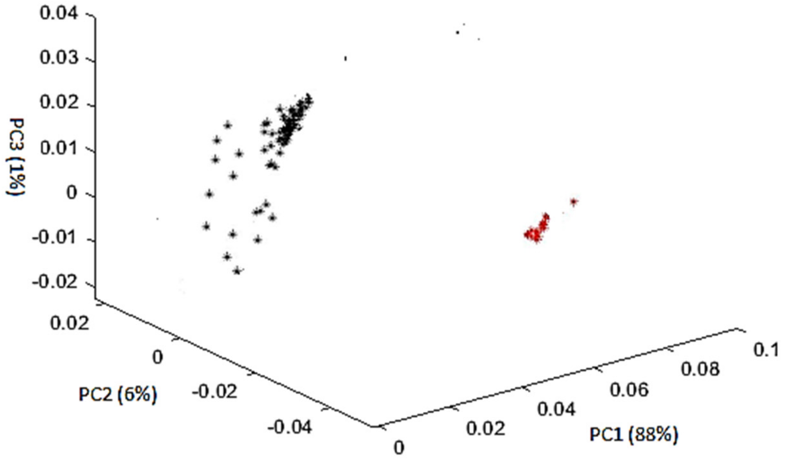

Figure S5. PCA plot obtained with the NIR spectra using the first derivative for Glycerol. Samples indicated with red stars are spiked samples and with black stars are non-spiked samples

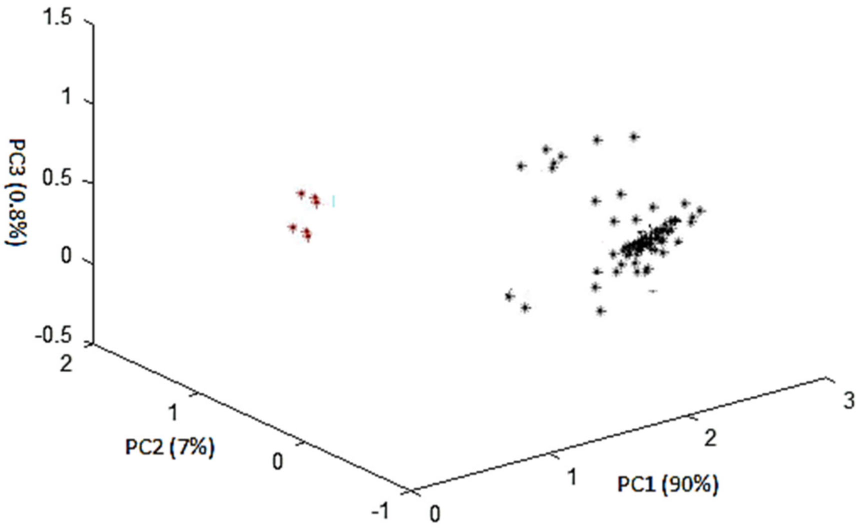

Figure S6. PCA plot obtained with the NIR spectra using the first derivative for Cocoa. Samples indicated with red stars are spiked samples and with black stars are non-spiked samples

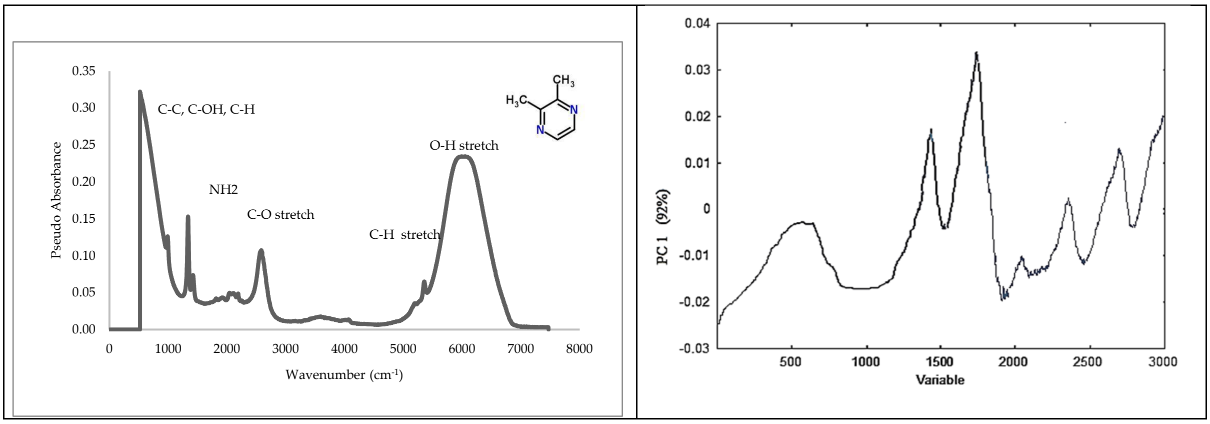

(a) (b)

Figure S7. (a) MIR spectrum of cocoa; (b) Loadings on PC1 highlighting the region important for discrimination for the cocoa

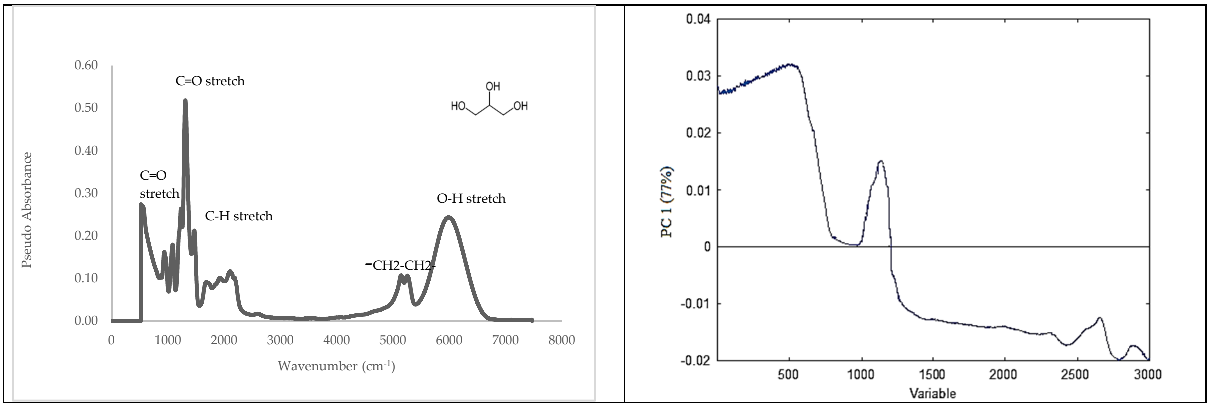

(a) (b)

Figure S8. (a) MIR spectrum of glycerol; (b) Loadings on PC1 highlighting the region important for discrimination for the glycerol

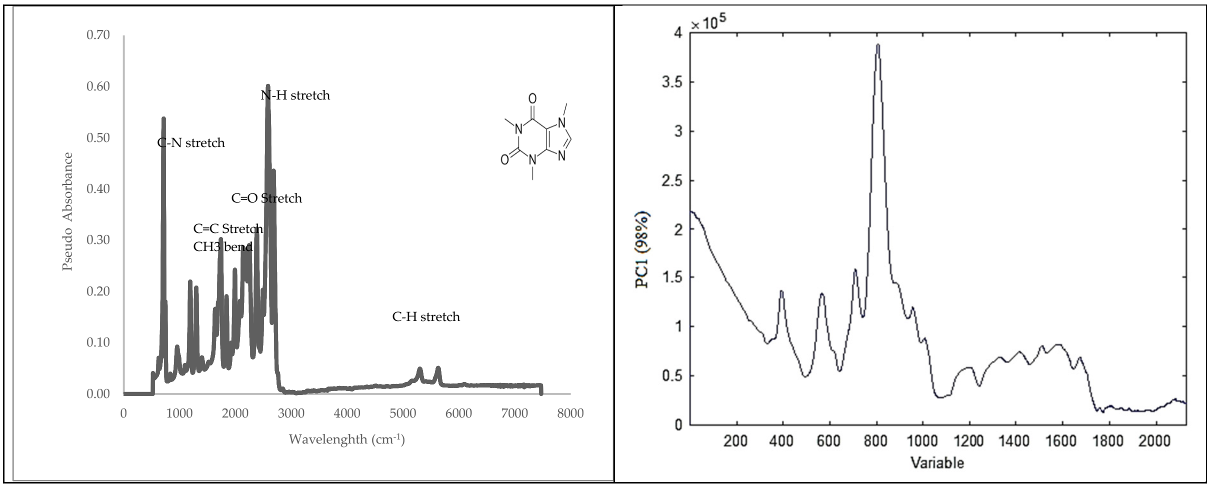

(a) (b)

Figure S9. (a) MIR spectrum of caffeine; (b) Loadings on PC1 highlighting the region important for discrimination for the caffeine

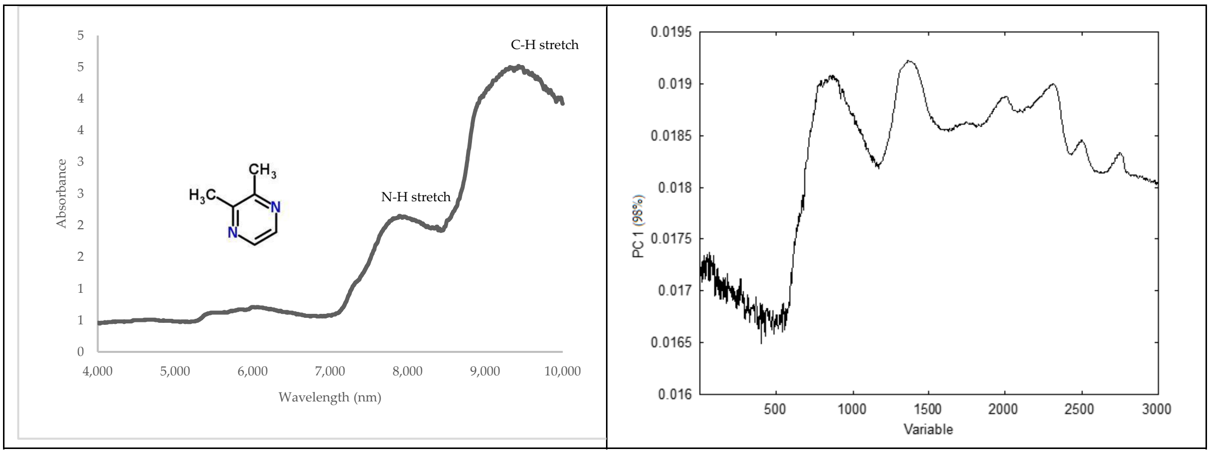

(a) (b)

Figure S10. (a) NIR spectrum of cocoa; (b) Loadings on PC1 highlighting the region important for discrimination for the cocoa

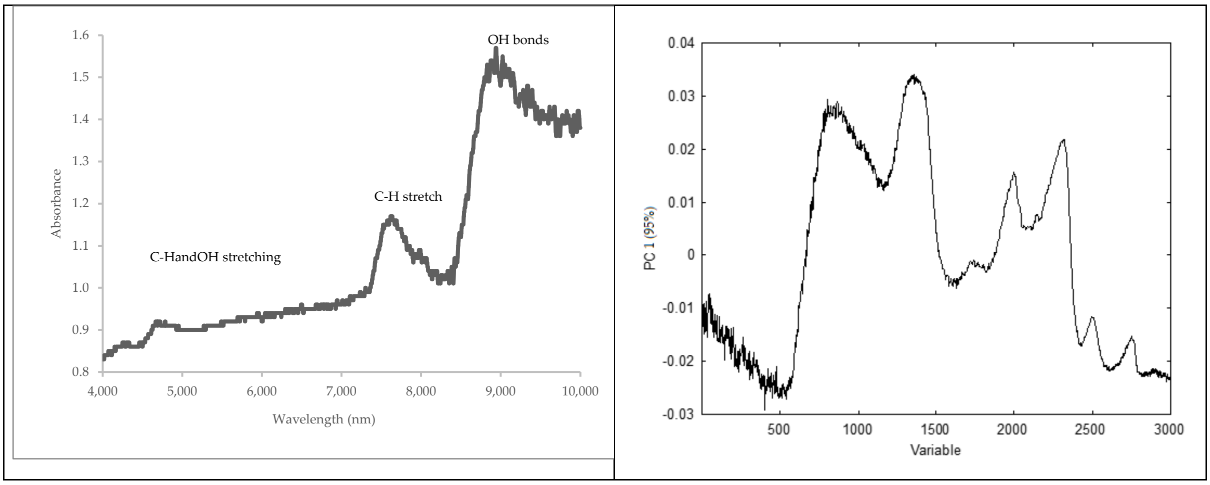

(a) (b)

Figure S11. (a) NIR spectrum of glycerol; (b) Loadings on PC1 highlighting the region important for discrimination for the glycerol

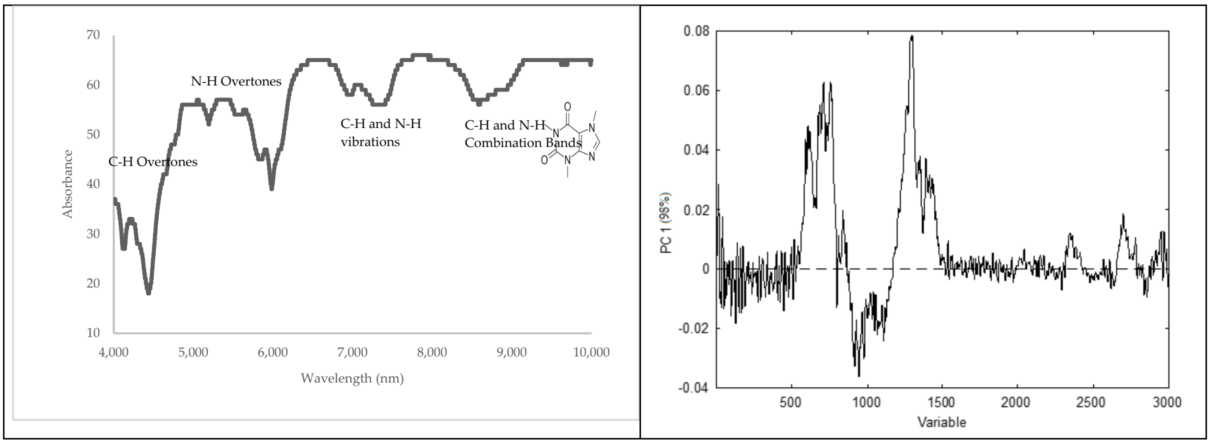

(a) (b)

Figure S12. (a) NIR spectrum of caffeine; (b) Loadings on PC1 highlighting the region important for discrimination for the caffeine

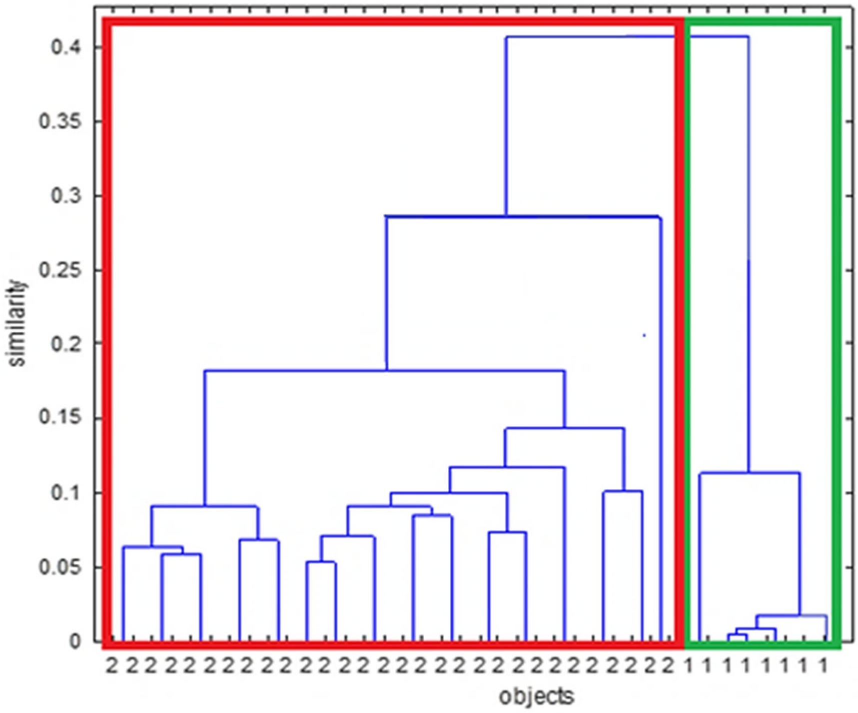

Figure S13. Dendrogram constructed via hierarchical clustering on MIR spectra for Caffeine. Samples indicated with 2 (red box) are spiked samples and with 1 (green box) are non-spiked samples

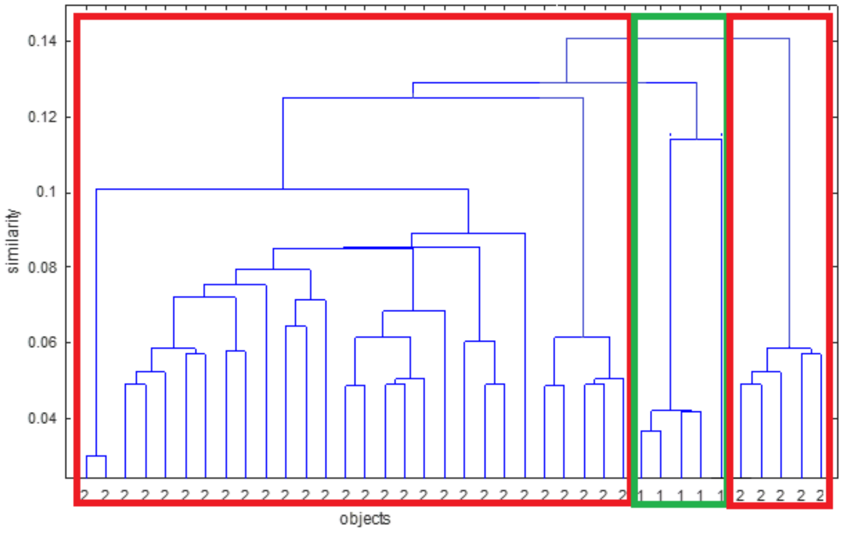

Figure S14. Dendrogram constructed via hierarchical clustering on NIR spectra for Caffeine. Samples indicated with 2 (red box) are spiked samples, and with 1 (green box) are non-spiked samples

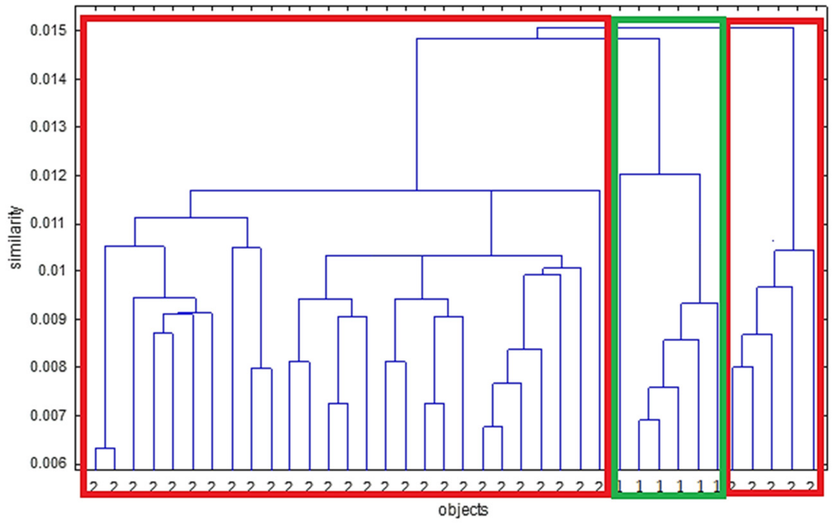

Figure S15. Dendrogram constructed via hierarchical clustering on MIR spectra for Glycerol. Samples indicated with 2 (red box) are spiked samples, and with 1 (green box) are non-spiked samples

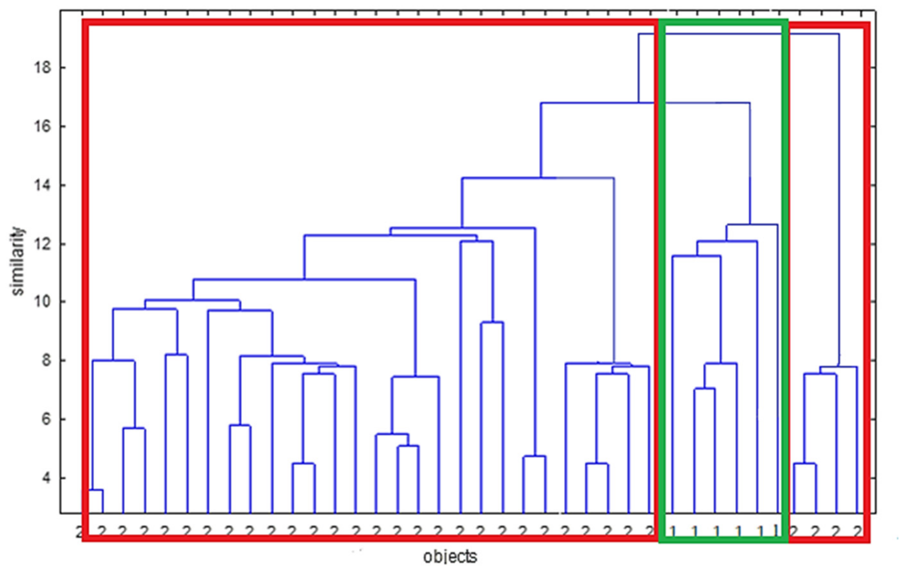

Figure S16. Dendrogram constructed via hierarchical clustering on NIR spectra for Glycerol. Samples indicated with 2 (red box) are spiked samples, and with 1 (green box) are non-spiked samples

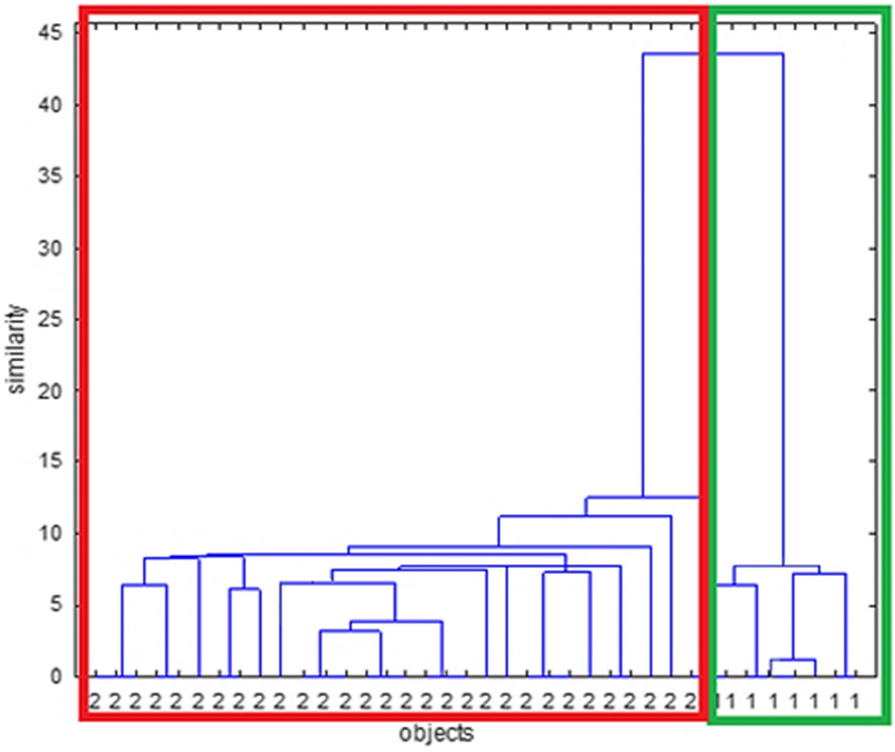

Figure S17. Dendrogram constructed via hierarchical clustering on MIR spectra for Cocoa. Samples indicated with 2 (red box) are spiked samples and with 1 (green box) are non-spiked samples

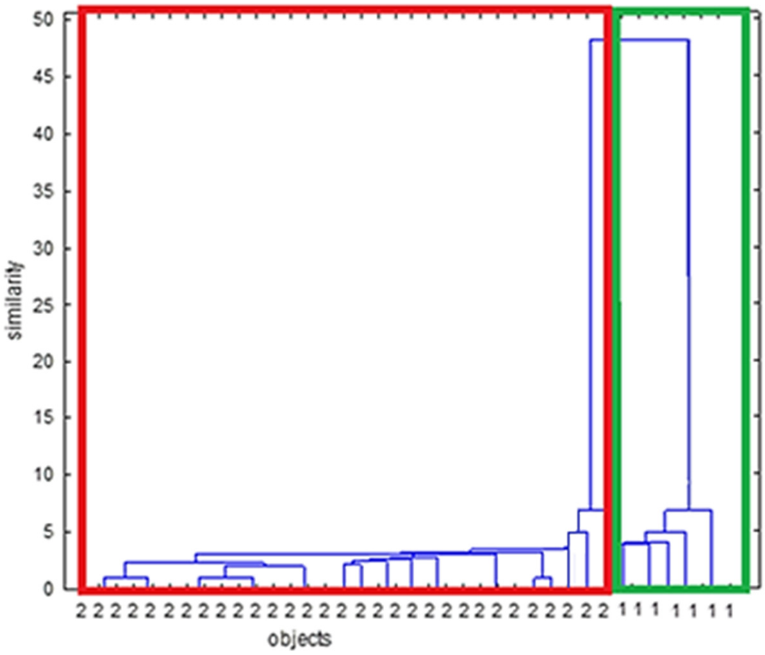

Figure S18. Dendrogram constructed via hierarchical clustering on NIR spectra for Cocoa. Samples indicated with 2 (red box) are spiked samples and with 1 (green box) are non-spiked samples

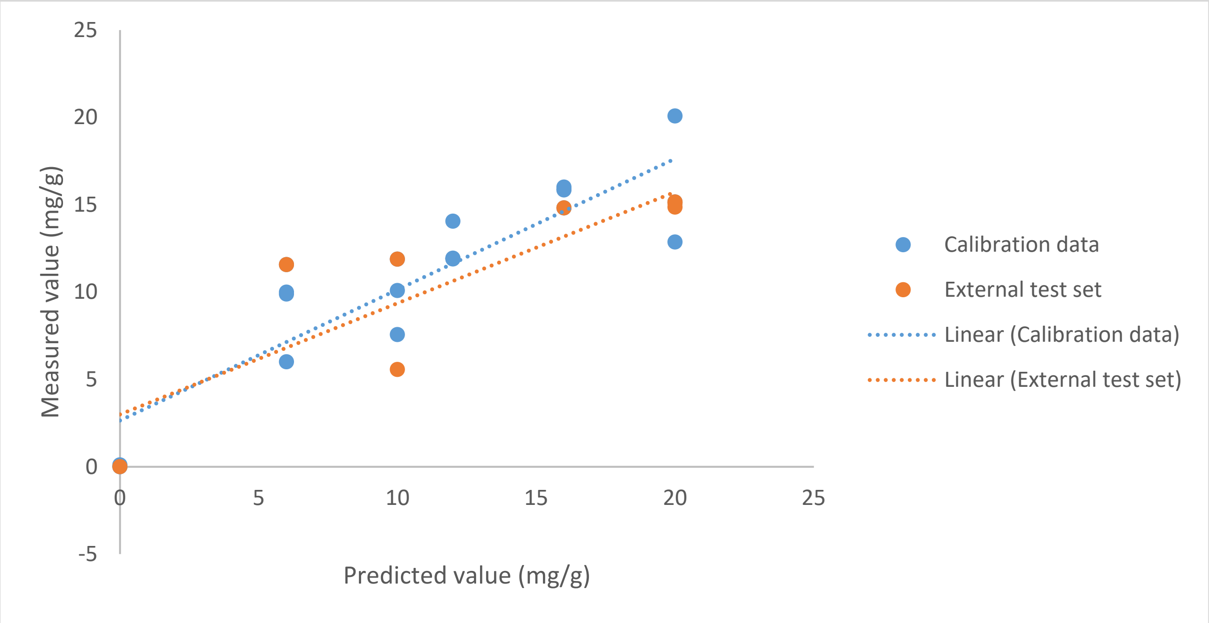

Figure S19. Insights into both the calibration accuracy and the predictive performance of the model for Cocoa (model based on MIR spectra using the 1st derivative as pretreatment method)

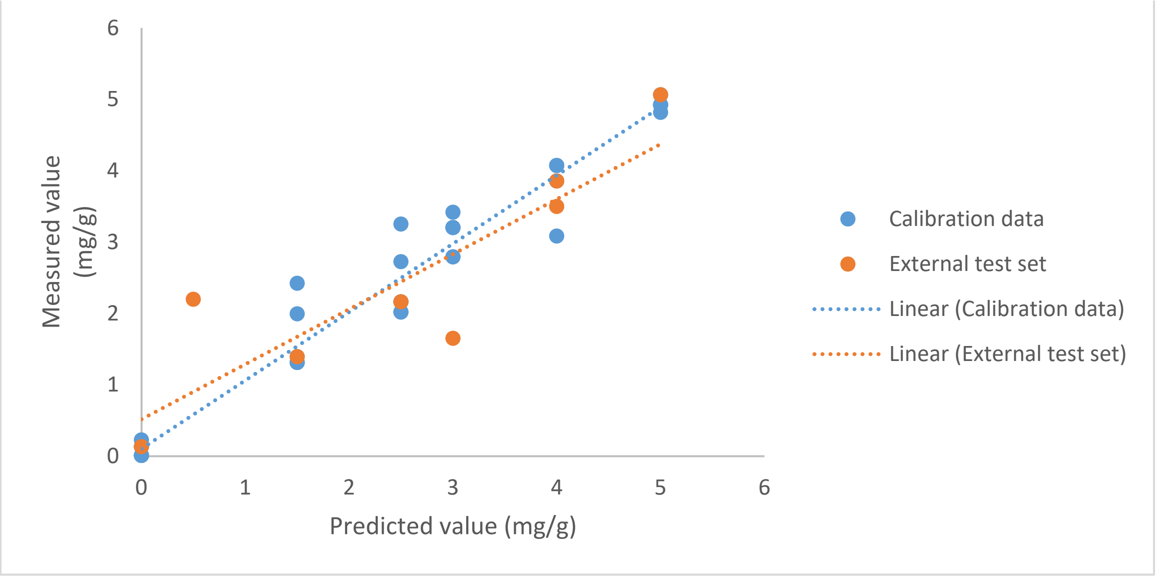

Figure S20. Insights into both the calibration accuracy and the predictive performance of the model for Menthol (model based on MIR spectra using the 1st derivative as pretreatment method)

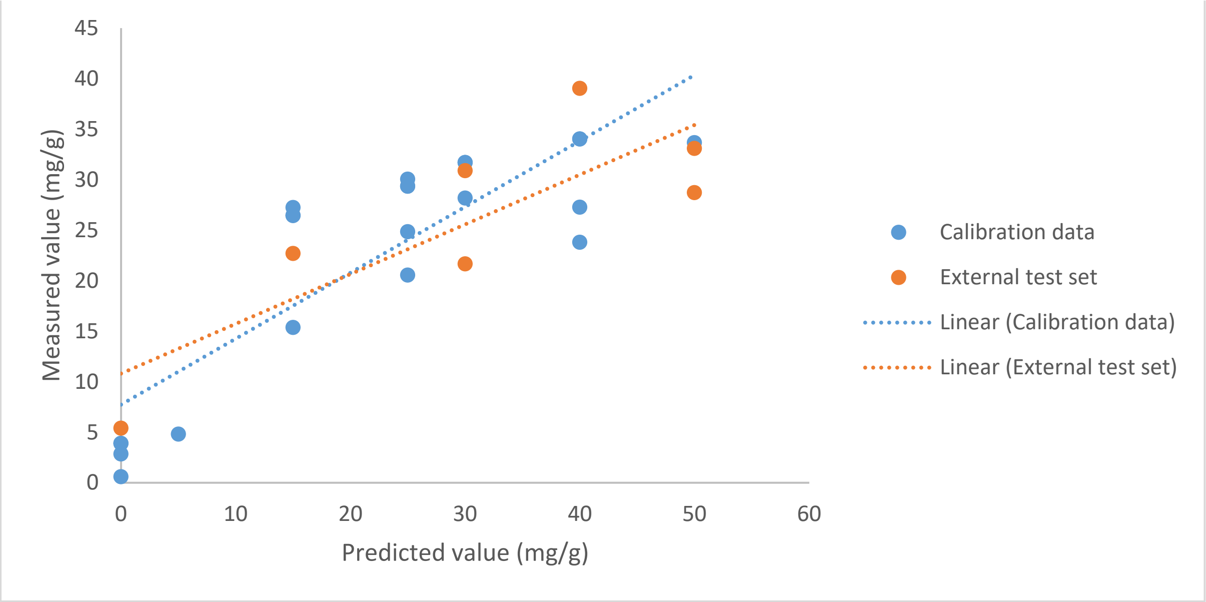

Figure S21. Insights into both the calibration accuracy and the predictive performance of the model for Glycerol (model based on MIR spectra using the 2nd derivative as pretreatment method)

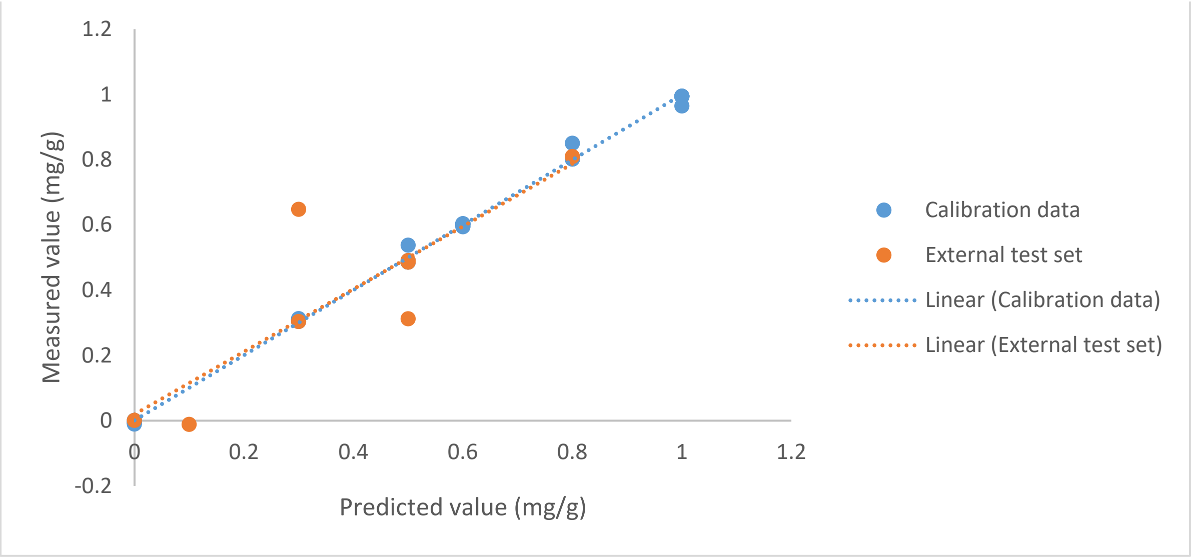

Figure S22. Insights into both the calibration accuracy and the predictive performance of the model for Caffeine (model based on NIR spectra using the 1st derivative as pretreatment method)

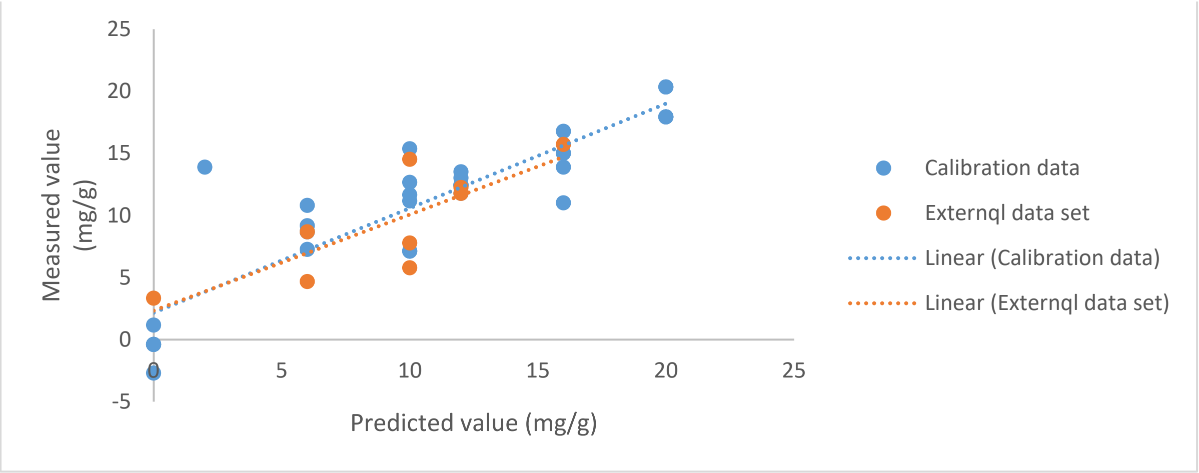

Figure S23. Insights into both the calibration accuracy and the predictive performance of the model for Cocoa (model based on NIR spectra using the SNV as pretreatment method)

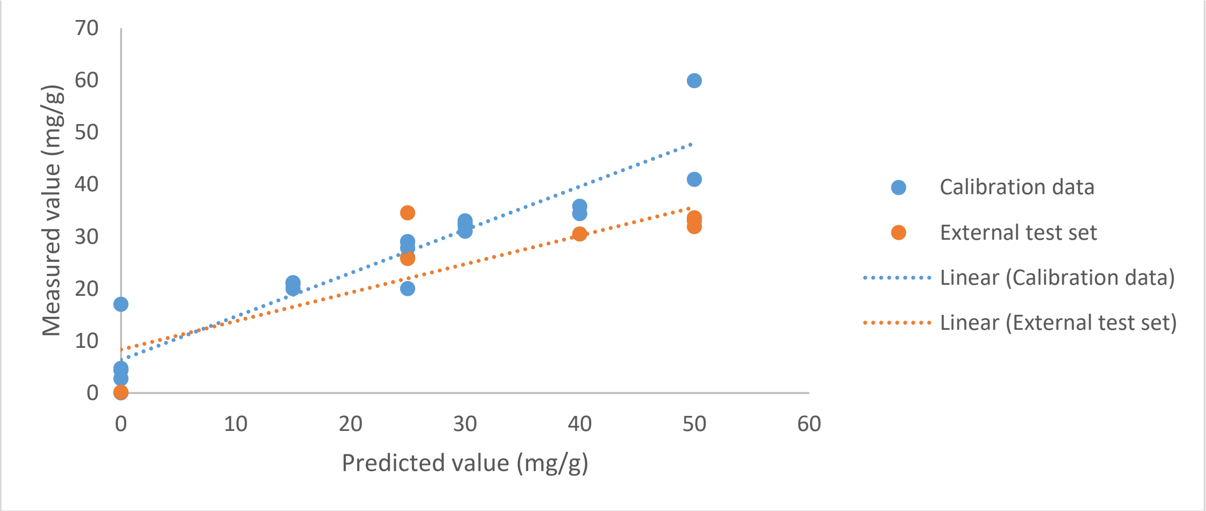

Figure S24. Insights into both the calibration accuracy and the predictive performance of the model for Glycerol (model based on NIR spectra using the 1st derivative as pretreatment method)

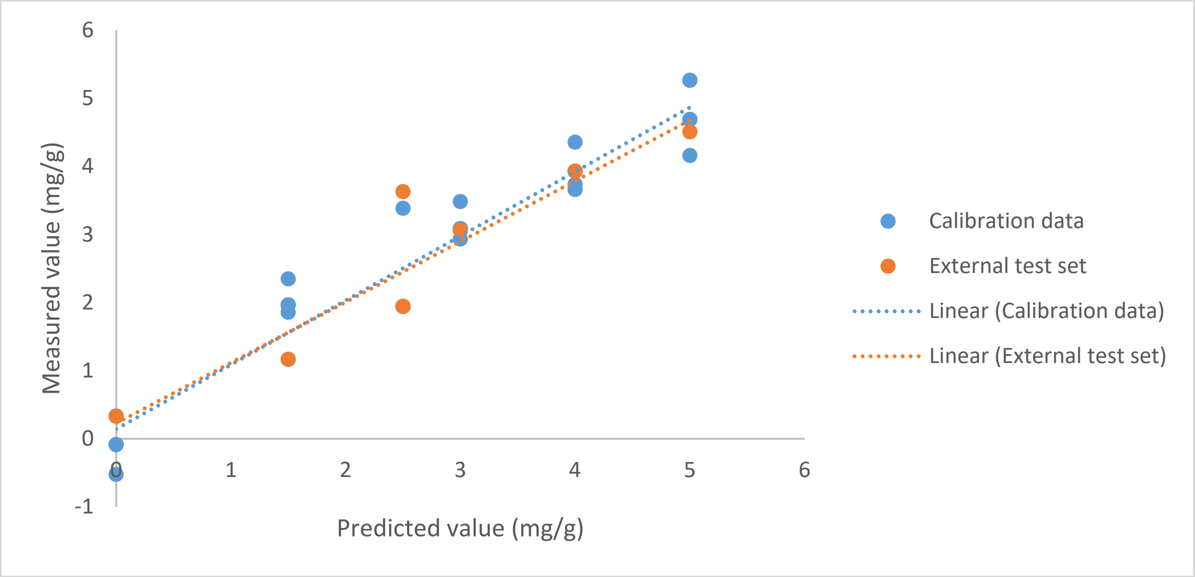

Figure S25. Insights into both the calibration accuracy and the predictive performance of the model for Menthol (model based on NIR spectra using the autoscaling as pretreatment method)

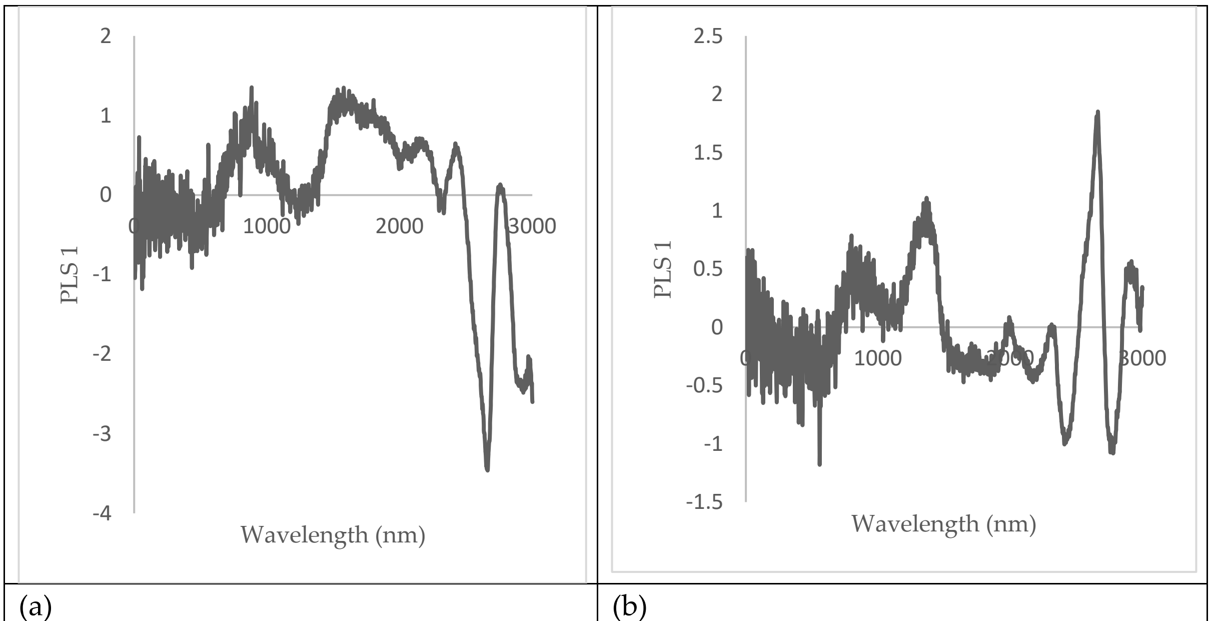

(a)	(b)

Figure S26. (a) PLS loading plot PLS1 for menthol (MIR) (b) PLS(DA) Loading plot PLS1 for menthol (MIR)

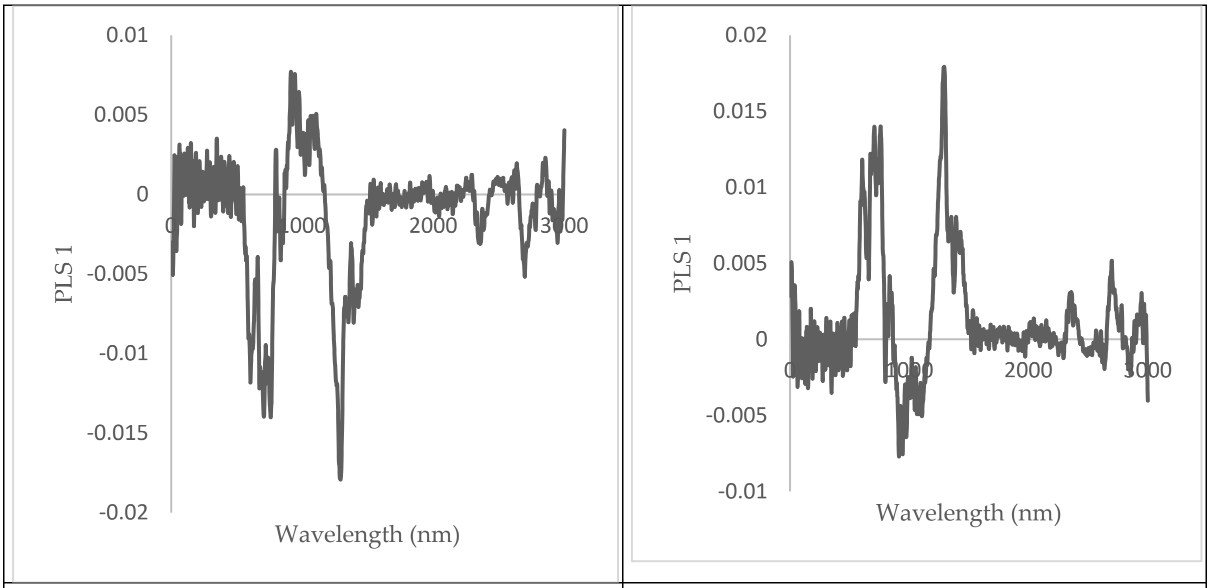

(a)	(b)

Figure S27. (a) PLS loading plot PLS1 for caffeine (MIR) (b) PLS(DA) Loading plot PLS1 for caffeine (MIR)

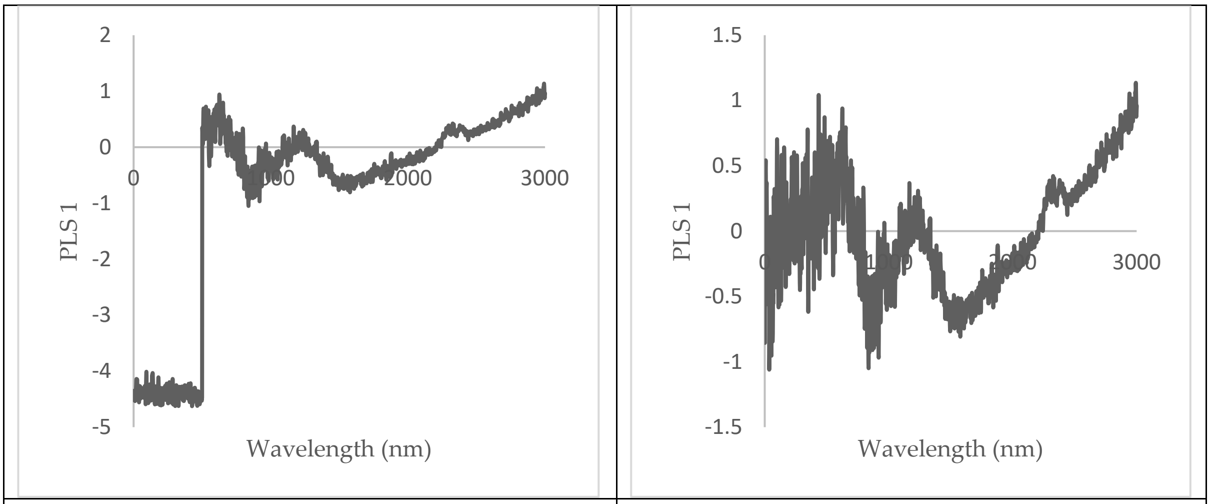

(a)	(b)

Figure S28. (a) PLS loading plot PLS1 for cocoa (MIR) (b) PLS(DA) Loading plot PLS1 for cocoa (MIR)

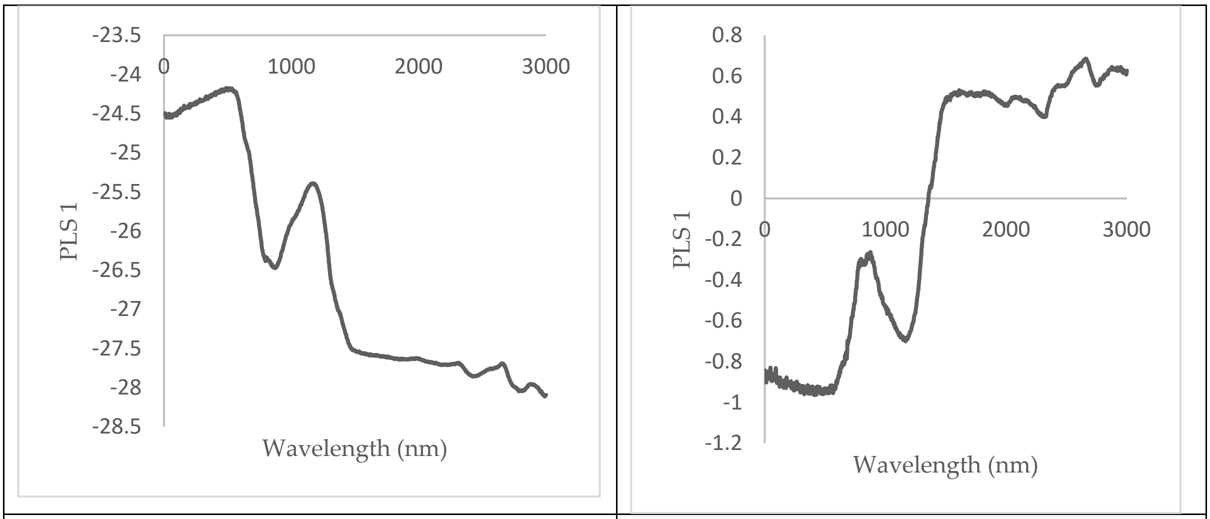

(a) (b)

Figure S29. (a) PLS loading plot PLS1 for glycerol (MIR) (b) PLS(DA) Loading plot PLS1 for glycerol (MIR)

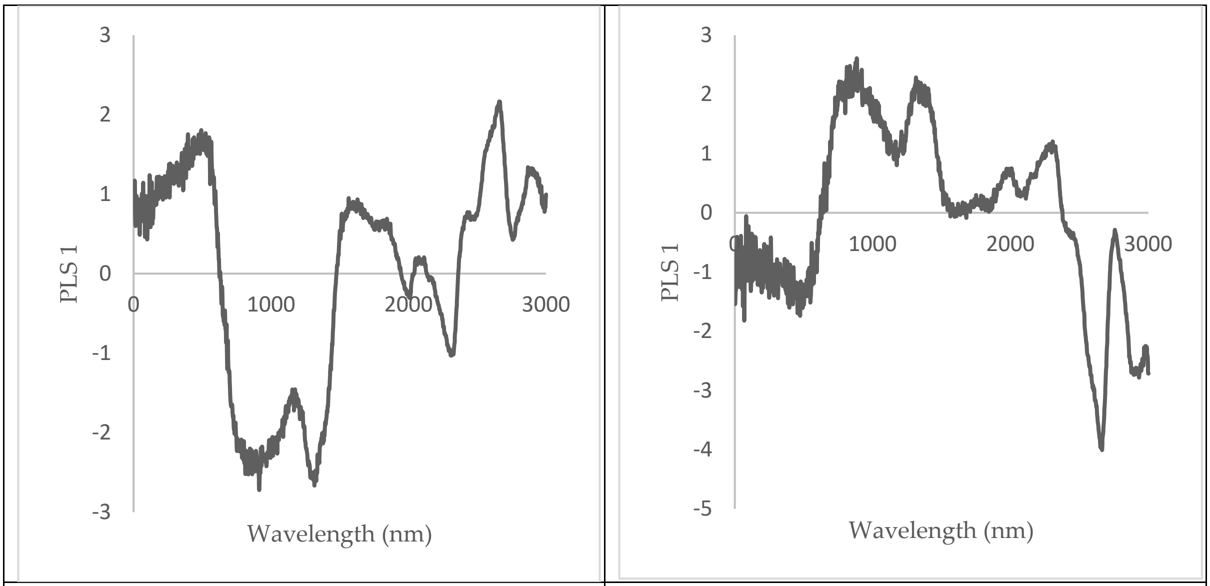

(a) (b)

Figure S30. (a) PLS loading plot PLS1 for glycerol (NIR) (b) PLS(DA) Loading plot PLS1 for glycerol (NIR)

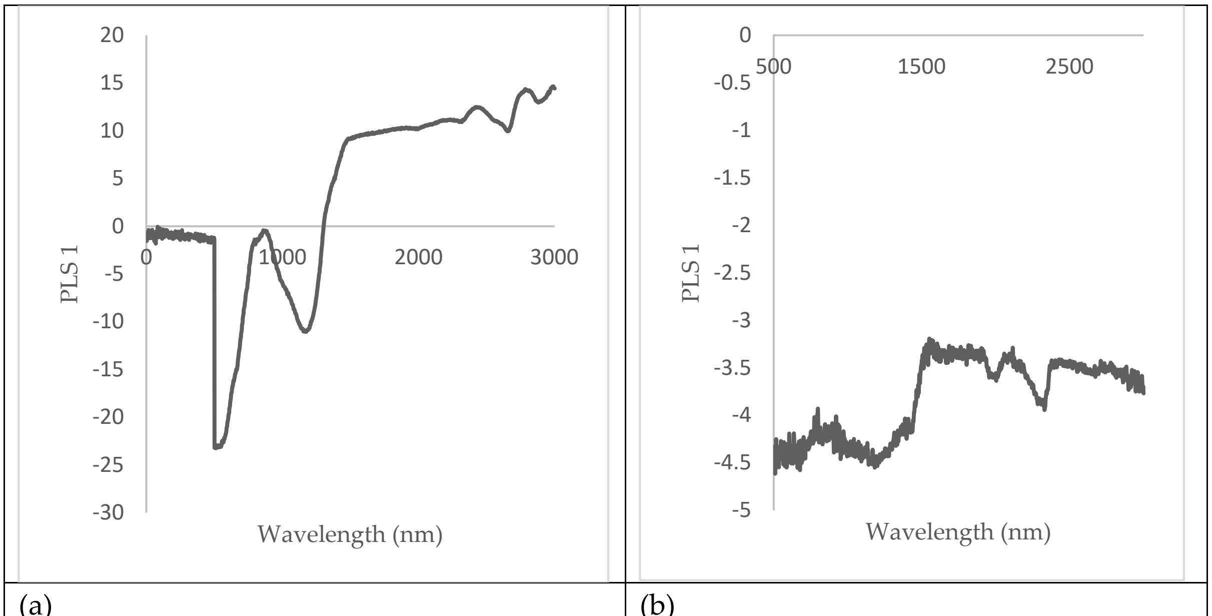

(a) (b)

Figure S31. (a) PLS loading plot PLS1 for cocoa (NIR) (b) PLS(DA) Loading plot PLS1 for cocoa (NIR)

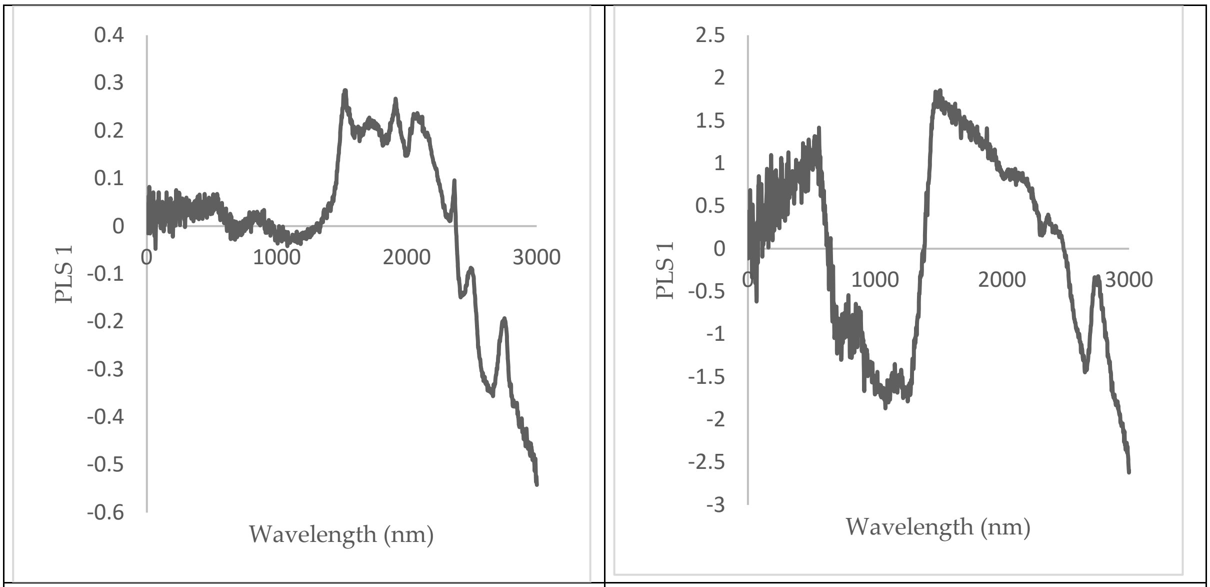

(a) (b)

Figure S32. (a) PLS loading plot PLS1 for caffeine (NIR) (b) PLS(DA) Loading plot PLS1 for caffeine (NIR)

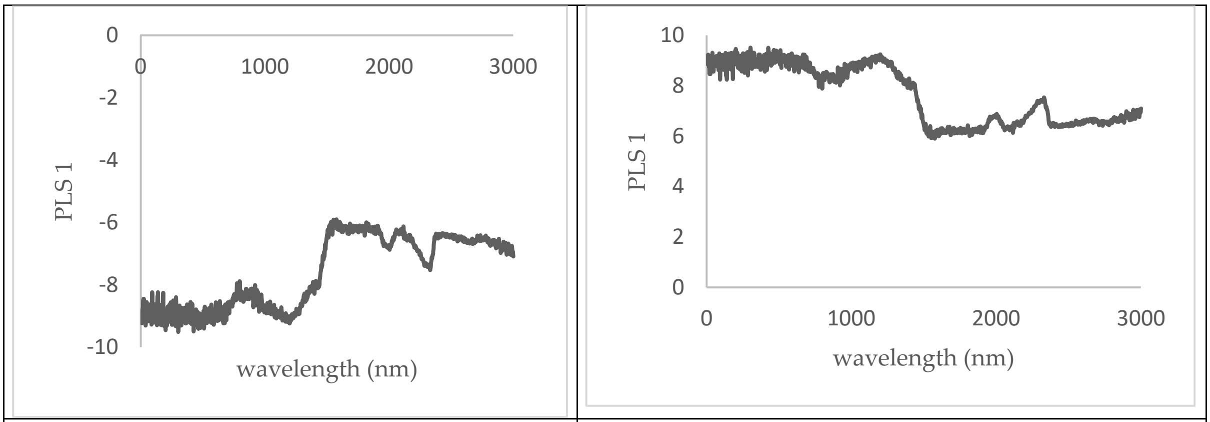

(a)	(b)

Figure S33. (a) PLS loading plot PLS1 for menthol (NIR) (b) PLS(DA) Loading plot PLS1 for menthol (NIR)

Table S1. GC-FID parameters to check the correctness of the spiked concentrations of menthol and glycerol for tobacco samples

**Headspace Parameters**	
Brand: Agilent 7697A	
**Temperature Settings:**	
Oven Temperature	85 °C
Loop Temperature	95 °C
Transfer Line Temperature	105 °C
**Timing Settings:**	
Vial Equilibration	10.00 min
Injection Duration	0.50 min
GC Cycle Time	70.00 min
**Vial and Loop Settings:**	
Vial Size	10
Vial Shaking	Level 5, 71 shakes/min with acceleration of 260 cm/s^2
Fill Mode	Default
Fill Pressure	20 psi
**GC Brand**	Agilent
**GC Summary**	
Run Time	60 min
Post Run Time	0 min
Oven	
Temperature	

Setpoint	On
(Initial)	40 °C
Hold Time	20 min
Post Run	0 °C
**Program**	
#1 Rate	10 °C/min
#1 Value	240 °C
#1 Hold Time	20 min
Equilibration Time	0 min
Max Temperature	280 °C
Maximum Temperature Override	Disabled
Slow Fan	Disabled
Back Volatiles Interface He	
Excluded from Readiness	***Excluded from Affecting GC's Readiness State***
Mode	Split
Heater	On 160 °C
Pressure	On 30.727 psi
Total Flow	On 32.24 mL/min
Septum Purge Flow	On 3 mL/min
Gas Saver	On 20 after 3.75 min mL/min
Split Ratio	16.2 :1
Split Flow	27.54 mL/min
Column	Agilent VF-5ms (CP9016)
Temperature range	-59 °C - 449 °C .
Dimensions	65 m x 250 µm x 0.25 µm

Table S2. HPLC-UV parameters to check the correctness of the spiked concentrations of caffeine for tobacco samples

**Parameter**	**Details**
**Brand**	Merck Hitachi System from Tokyo Japan
**Column type**	Zorbax Eclipse Plus C18
**Pore size**	5 µm
**Internal diameter**	4.6 mm
**Column length**	150 mm
**Phase**	Reverse phase – ODS
**Flow rate**	1 mL/min (constant)
**Column temperature**	40 °C

**Detector**	UV detector set at 275 nm
**Mobile phase**	Water - Methanol (60:40), both HPLC grade
**Sample injection volume**	10 μL
